# Genetic Variation in the Nuclear and Organellar Genomes Modulates Stochastic Variation in the Metabolome, Growth, and Defense

**DOI:** 10.1371/journal.pgen.1004779

**Published:** 2015-01-08

**Authors:** Bindu Joseph, Jason A. Corwin, Daniel J. Kliebenstein

**Affiliations:** 1 Department of Plant Sciences, University of California, Davis, Davis, California, United States of America; 2 DynaMo Center of Excellence, University of Copenhagen, Frederiksberg, Denmark; The University of North Carolina at Chapel Hill, United States of America

## Abstract

Recent studies are starting to show that genetic control over stochastic variation is a key evolutionary solution of single celled organisms in the face of unpredictable environments. This has been expanded to show that genetic variation can alter stochastic variation in transcriptional processes within multi-cellular eukaryotes. However, little is known about how genetic diversity can control stochastic variation within more non-cell autonomous phenotypes. Using an Arabidopsis reciprocal RIL population, we showed that there is significant genetic diversity influencing stochastic variation in the plant metabolome, defense chemistry, and growth. This genetic diversity included loci specific for the stochastic variation of each phenotypic class that did not affect the other phenotypic classes or the average phenotype. This suggests that the organism's networks are established so that noise can exist in one phenotypic level like metabolism and not permeate up or down to different phenotypic levels. Further, the genomic variation within the plastid and mitochondria also had significant effects on the stochastic variation of all phenotypic classes. The genetic influence over stochastic variation within the metabolome was highly metabolite specific, with neighboring metabolites in the same metabolic pathway frequently showing different levels of noise. As expected from bet-hedging theory, there was more genetic diversity and a wider range of stochastic variation for defense chemistry than found for primary metabolism. Thus, it is possible to begin dissecting the stochastic variation of whole organismal phenotypes in multi-cellular organisms. Further, there are loci that modulate stochastic variation at different phenotypic levels. Finding the identity of these genes will be key to developing complete models linking genotype to phenotype.

## Author Summary

Systems biology is largely based on the principal that the link between genotype and phenotype is deterministic, and, if we know enough, can be predicted with high accuracy. In contrast, recent work studying transcription within single celled organisms has shown that the genotype to phenotype link is stochastic, i.e. a single genotype actually makes a range of phenotypes even in a single environment. Further, natural variation within genes can lead to each allele displaying a different phenotypic distribution. To test if multi-cellular organisms also display natural genetic variation in the stochastic link between genotype and phenotype, we measured the metabolome, growth and defense metabolism within an Arabidopsis RIL population and mapped quantitative trait loci. We show that genetic variation in the nuclear and organeller genomes influence the stochastic variation in all measured traits. Further, each trait class has distinct genetics underlying the stochastic variance, showing that there are different mechanisms controlling the stochastic genotype to phenotype link for each trait. Further work is necessary to identify the mechanisms underpinning the stochastic nature of the genotype to phenotype link.

## Introduction

The link between genotype and phenotype is often considered to be deterministic such that a single genotype functions to yield a specific phenotypic value. This deterministic relationship is a central tenet of the desire to develop predictive models allowing an organism's phenotype to be forecasted upon knowing its specific genotype. This deterministic hypothesis is supported by research showing that cells limit stochastic noise/variance in genetic, metabolic, and signaling networks through network topology, a characteristic that is known as network robustness [1]-[6]. This robustness is an inherent property of genetic networks. In evolutionary theory, robustness is predominantly described as canalization wherein genes function to minimize the variance (maximize the robustness) of a phenotype [7]–[11]. A well-studied example of genetic control over variance for diverse phenotypes is the heat-shock protein 90 which plays a major role in canalizing existing natural variation [12]–[14].

While a deterministic link between genotype and phenotype is the most frequently studied aspect of evolution and genetics, there is growing research showing the potential evolutionary benefit of a stochastic link between genotype and phenotype. A stochastic link between phenotype and genotype allows an individual genotype to generate a range of phenotypes within a specific environment and causes the portfolio effect wherein the fitness of a specific genotype is determined by the range of phenotypes that it can obtain [15]. In some bacterial settings, stochastic switching of the genotype-to-phenotype link is the evolutionary optimal response to rapid unpredictable environmental fluctuations [16]–[20]. Similarly in single-celled and multicellular eukaryotes, there is beginning to be studies finding polygenic natural variation that determines stochastic noise of gene expression [21]–[25]. This includes *Arabidopsis thaliana* loci that are known to be under natural selection suggesting that the stochastic aspects of these loci may impart an evolutionary benefit [24], [26], [27]. One possible evolutionary benefit of this phenomenon to higher-eukaryotes is that stochastic noise in defense phenotypes can delay the evolution of counter-resistance in biotic pests [28], [29]. Thus, there is just beginning to be an appreciation of genetic variation controlling stochastic noise in eukaryotic gene expression, which may play a beneficial role in the evolution of these organisms [12]–[14], [20] [21]–[25].

Similar to transcriptional networks, metabolic networks are thought to be highly structured to maximize deterministic relationships and minimize stochastic variance that could disconnect pathways and potentially generate toxic intermediates [30]. Metabolic robustness is thought to arise from the fact that metabolism is highly interconnected with numerous feedback loops and parallel pathways involving enzymes encoded by both the nuclear and organellar genomes in eukaryotes [31]. This hypothesis was supported by a recent modelling approach where only a few enzymes were predicted to influence stochastic variation in the whole metabolic network [32]. In contrast, a different modelling effort found that stochastic noise can arise in local areas of a metabolic network without spreading throughout the system. This suggests that stochastic variation in the metabolome could be caused by numerous independent loci. [33] However, a lack of empirical evidence on the level or presence of genetically-controlled stochastic variation within metabolism prevents a direct comparison of these two models [24].

To empirically measure the potential for genetic variation to control stochastic variation within the metabolomic network, we measured metabolome variation in a recombinant inbred line (RIL) population of *Arabidopsis thaliana*. Arabidopsis is a key organism in the study of complex traits including the genetic programming of stochastic variation through the use of systems biology and quantitative genomics approaches [24], [34]–[41]. Additionally, Arabidopsis has been a model system to study the quantitative basis of metabolomic variation in a number of structured and unstructured populations [42]–[44]. Combined with extensive whole genome sequence of natural accessions, this provides the ability to rapidly develop and test hypotheses, as well as find causal genes underlying specific loci of interest [45]–[49]. Finally, there are a large number of existing homozygous populations to enable this analysis [50]. This makes Arabidopsis an ideal system to search for the genetic and molecular basis of complex phenotypes, such as stochastic noise, in higher organisms.

Using a replicated, randomized sampling design, we measured metabolome variation in the Kas x Tsu RIL population and compared the quantitative genetics for average metabolite accumulation versus the stochastic variation [24], [51]. The independently replicated analysis of CV allows us to separate stochastic variance from non-additive variance affecting the mean. This is in contrast to recent efforts to map variance QTLs using un-replicated data which conflates the two [52]–[55]. To test if defense or growth traits may differentially affect the link between CV and mean, we also measured the variation in growth and defense chemistry [51]. As found in a previous analysis of the Arabidopsis transcriptome, stochastic variation showed a higher heritability than that for variation in the average phenotype. As found for the transcriptome, there were differences in the genetics controlling the stochastic variation and average phenotypes. In support of ecological/bet-hedging theory, defense chemistry showed more QTLs of larger effect for stochastic variance than those found for growth or primary metabolism. Importantly, the genetic variation within the organelle had a widespread effect on the stochastic variation in primary metabolism with discrete impacts that differed from the organelle effect on the average metabolome. Thus, natural variation has widespread effects on the stochastic variation of growth and metabolism involving both the nuclear and organellar genomes. Future work will identify if the genetic basis of the average and stochastic variation are caused by similar or dissimilar mechanisms.

## Results

### Heritable stochastic noise in plant growth and metabolite phenotypes

To test if genetic variation affects stochastic noise in the metabolome and growth of the higher plant *Arabidopsis thaliana*, we used a previous analysis of quantitative variation of the average metabolism and growth within the Kas x Tsu RIL population [51], [56]. A total of 559 metabolomic, 19 chemical defense and 5 growth traits were measured in this population with replicated independent experiments providing replication on both the average and standard deviation of each phenotype. Using this data, we obtained the coefficient of variance (CV) for each phenotype in each experiment for each RIL. This was done by dividing the standard deviation of the phenotype within an experiment by its mean within that same experiment. CV is an appropriate comparative measure of genotypic stochastic noise as it is a dimensionless measure of variation allowing us to perform the ensuing analysis [16], [21]. All per line CV measures were compared to the previously published analysis of the average phenotypes for the same traits [51], [56].

**Figure 1:**
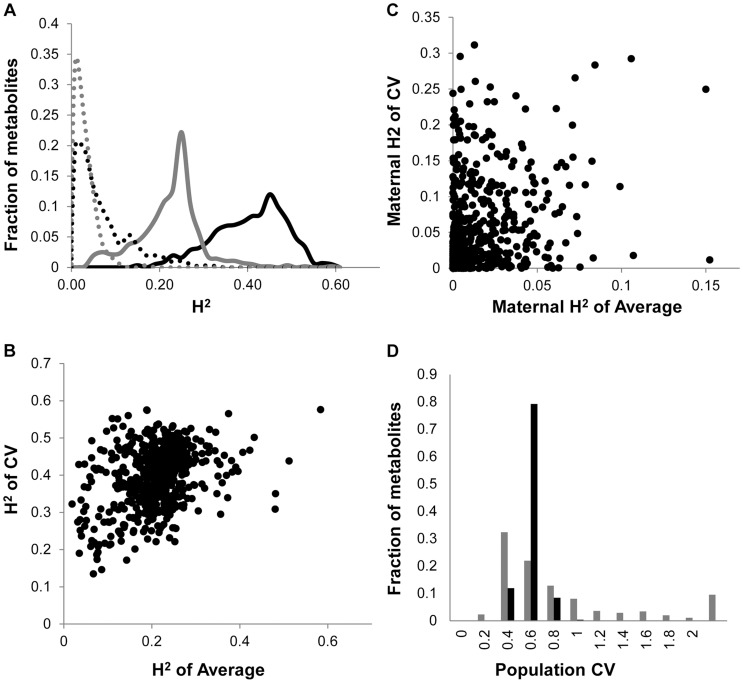
Comparison of CV and Average metabolome genetics in Kas x Tsu. A. Comparison of estimated metabolite heritability's using each metabolites CV (black) and average (grey) phenotype across Kas x Tsu RIL populations. A frequency plot shows the estimated heritability's ascribed to the nuclear (solid lines) and organellar (dotted) genomes across all the metabolites. B. Scatter plot of genotypic heritability for 559 metabolites where both average and CV heritability could be estimated in the Kas x Tsu RIL population. C. Scatter plot of maternal heritability for 559 metabolites where both average and CV heritability could be estimated in the Kas x Tsu RIL population. D. Distribution of genetic variation controlling the CV (black) and average (grey) metabolic phenotypes within the population are shown as the genetic coefficient of variance.

As previously found using the Arabidopsis transcriptome, the heritability for the metabolite CV was higher than that for the average metabolite accumulation (Fig. 1A and S1 Table) [24]. In addition to the metabolome, both growth and defense chemistry also showed increased heritability for per line CV in comparison to the average (S1 Fig.). Comparing the heritability of per line CV and average across all the metabolites showed that there was no correlation between these two values (Fig. 1B). Similarly, there is no correlation between mean and CV for the metabolites across all the RILs (S2 Fig.). Thus, per line CV is not being driven simply by variation in the level of the average phenotype within this dataset but is instead an independent output of the genetic variation in comparison to the average metabolite accumulation. Similar to the transcriptome, the range of metabolite CV across the RILs was less than that found for the average metabolite accumulation (Fig. 1D).

### Heritable stochastic noise in plant growth and metabolite phenotypes caused by cytoplasmic genetic variation

The Kas x Tsu population is a reciprocal population that allows us to measure the relative contribution of the nuclear and organellar genomes to any resulting phenotypes by using the maternally inherited organellar genomes as a single marker [57]. Because these RILs are in their F10 generation due to bulking in our lab and all seed mothers for the RILs for this experiment were grown together and harvested at the same time, we are largely focusing on maternal effects due to the genetic variation in the organelles. Thus, we used a linear model to estimate the contribution of the organellar genome variation to heritability of per line CV across the metabolome. This showed that the organellar genomes contributed 5.4%±0.2% heritability with a max of 31% heritability for metabolites (Fig. 1A and S1 Table). This organellar genome heritability for per line metabolite CV was significantly higher than that found for average metabolite accumulation (Fig. 1A) [51]. Again, there was no correlation between the heritability of per line CV and average driven by the organellar genome across the metabolites (Fig. 1C). This suggests that as with the nuclear genome, the effect of the organellar genomic variation on CV is separate from that of the effect on average metabolite accumulation (Fig. 1C). In contrast to the metabolome, the cytoplasm had similar heritable effects on the CV of growth and defense chemistry as that found for the average (S1 Fig.). Thus, the genetic variation in the organelles of Arabidopsis can heritably influence per line CV of plant metabolism, growth, and defense chemistry.

### Genetic variation in CV and average alter different metabolite functionalities

**Figure 2:**
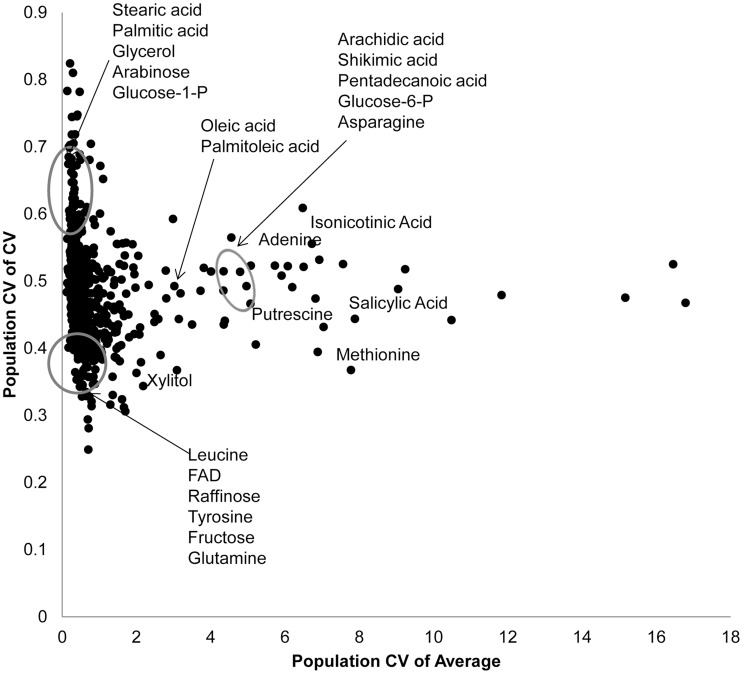
Genetic variation in Kas x Tsu for CV and average targets specific metabolites. Using both per RIL CV and average for the 559 metabolites where CV could be measured, we estimated the genetic coefficient of variation across the population. This allows us to visualize which metabolites show a high level of genetic variation affecting per RIL CV (y axis) and average (x-axis) for each metabolite. The known metabolites labeled and encircled at the bottom left of the graph are in the bottom 10^th^ percentile of the genetic coefficient of variance for both the mean and CV. The known metabolites encircled at the top left of the graph are in the top 10^th^ percentile of for CV but the bottom for mean. The remaining labeled metabolites are in the top 10^th^ percentile for population average with close to average population CV. Only known metabolites are labeled.

Using per line CV and average metabolite accumulation across all the RILs, we can obtain the genetic coefficient of variation across the population (Population CV), which describes the range of variation for that trait across the RILs. Correlating range of variation across the population for CV and average using all the metabolites showed that there was a continuous range of variation in the relationship between population variation in mean and CV. To test if there might be some biological insight within these distributions, we focused on the metabolites whose population variation that were in the top 5% or bottom 5% of the metabolites for either mean or CV. This allowed us to define three groupings (Fig. 2). One grouping was characterized by metabolites where the population CV is in the top 5% of all metabolites but the variation of average for these same metabolites is within the bottom 5% (Top left of Fig. 2). This included lipids, such as Steric and Palmitic acid, as well as energy sources into lipid metabolism, like glycerol and Glucose-1-P. Contrastingly, a set metabolites that consist predominantly of amino acids and sugars, were in the bottom 5% of all metabolites for population variation in both CV and average (Bottom left of Fig. 2). This would suggest that these metabolites are constrained or robust within this population. There was also a set of metabolites whose average accumulation was within the top 5% of all metabolites yet their CV was not an outlier (Right of Fig. 2). This included stress inducible metabolites like Putrescine, Isonicotinc acid, Salicylic acid, Shikimic acid and Methionine (Fig. 2). These metabolites should be the more sensitive to micro-environmental variation in stress than the other compounds. The fact that these stress sensitive metabolites only have intermediate variation in CV within this population further suggests that we are measuring genetic diversity in CV rather than any micro-environmental effect.

### Mapping QTL for metabolite CV

**Figure 3:**
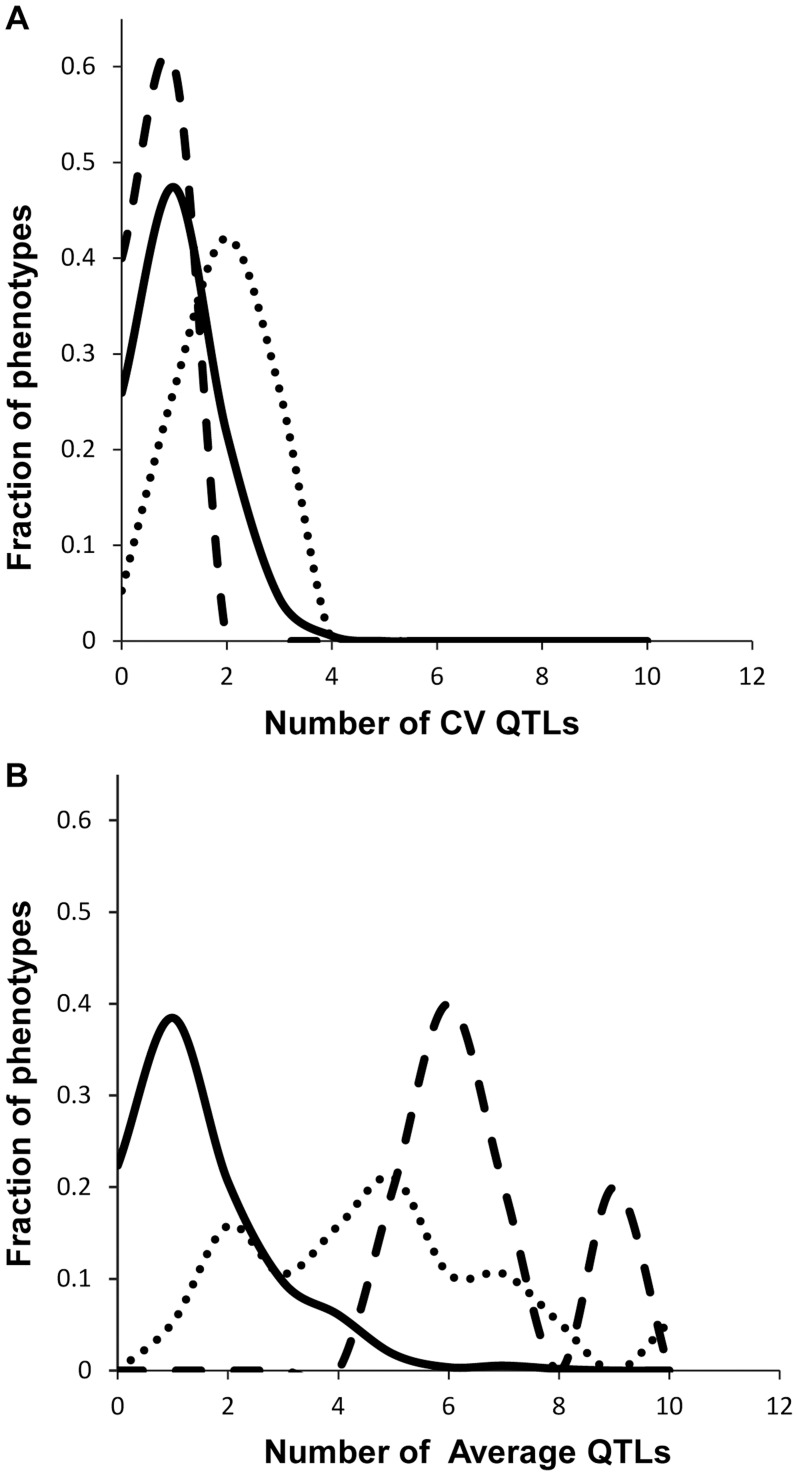
Comparison of QTL detection across phenotype classes. Shown is the frequency of metabolite, defense or growth phenotypes that detected a given number of nuclear genome QTLs. Solid lines for metabolites, dotted lines for defensive glucosinolates and dashed lines for growth. A. Shows the number of QTLs for the CV phenotype. B. Shows the number of QTLs for the average phenotype.

**Figure 4:**
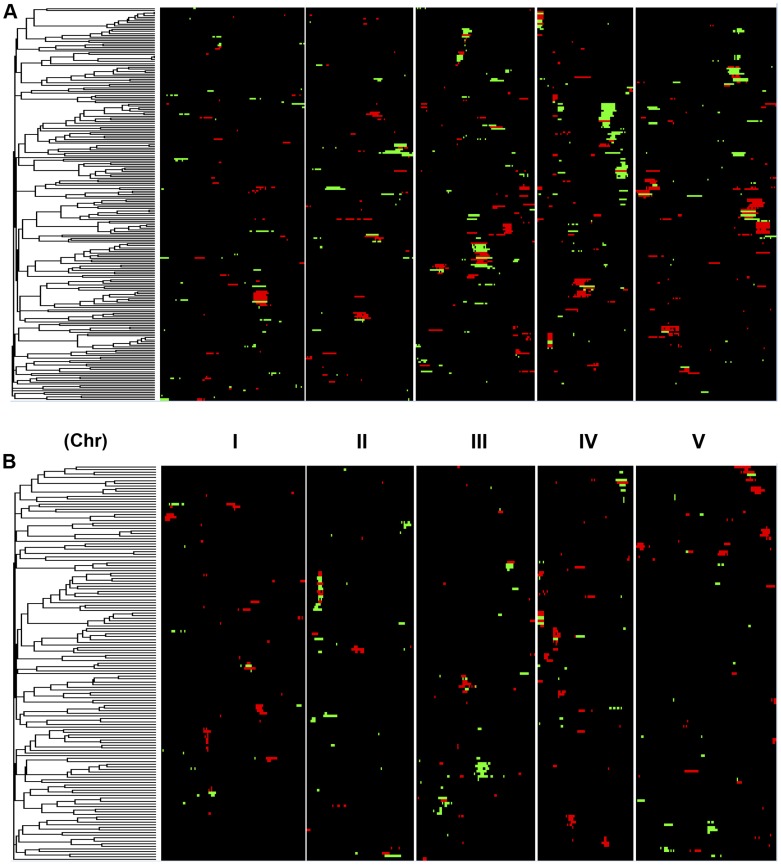
Genetic architecture of metabolite QTLs across the Kas x Tsu Genome. Heat map showing the location and effect of loci detected for metabolite average with LOD scores above the permuted LOD threshold 2, across the five chromosomes. Red indicates a positive effect of the Kas allele, while green indicates a positive effect of the Tsu allele. Vertical white lines separate the chromosomes (I to V from left to right). Clustering on the left is based on the absolute Pearson correlation of QTL effects across all significant loci for each metabolite. A. QTLs identified for average metabolite accumulation. B. QTLs identified for CV in metabolite accumulation.

We obtained the average and per line CV for each metabolite for each RIL from the linear model used to estimate heritability. We used these values to map QTLs for both phenotypes across all 559 metabolites for all 271 RILs with fully replicated data. This analysis identified on average 3 QTL for 434 metabolites using the average accumulation and 1.75 QTL for 414 different metabolites using the per line CV (Fig. 3 and S3 Table) [51], [56]. There was no observable correlation in the number of CV or average QTLs across the metabolites nor in the effect of overlapping QTLs (S3 Fig.). This decrease in QTL identification for per line CV is similar to previous analysis using transcriptomic variation in a different Arabidopsis population [24]. The mean effect of each identified metabolite average QTL was 22% in comparison to 17% for metabolite CV QTL, which also agrees with what was previously found using the transcriptome (Figs. 3 and 4) [24]. The fact that per line CV has higher heritability with fewer detectable QTL of lower effect size than the average phenotype suggests that per line CV likely has a more polygenic genetic basis than that controlling the average metabolite accumulation [58], [59].

**Figure 5:**
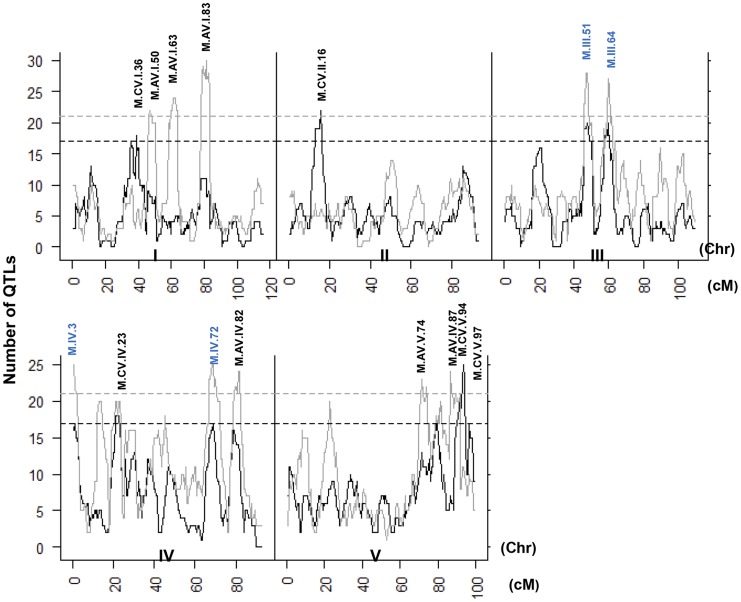
QTL hotspots for metabolite average and CV. The number of metabolites for which a QTL was detected within a 5 cM sliding window is plotted against the genetic location of the metabolite QTLs in cM. Metabolite average QTLs are shown in grey and CV in black. The permuted threshold (*P* = 0.05) for detection of a significant metabolite hotspot is 21 QTLs for metabolite average and 17 for metabolite CV. Hotspots are labeled above the respective locus with AV representing Average and CV representing CV followed by chromosome number and cM position. The hotspots labeled in blue are detected for both average and CV.

A comparison of the QTL maps across all the metabolites showed that the patterns of loci were not identical (Fig. 4). This suggested that there might be different loci controlling the average and CV of metabolite accumulation in these RILs. Overlapping the QTL hotspots identified using the average and CV metabolic phenotypes across all metabolites showed that this was in fact the case (Fig. 5). There were QTL hotspots specific for either the average or per line CV of metabolite accumulation. There were five hotspots statistically unique to per line CV. For example, the QTL on Chromosome II (M.CV.II.15) was entirely linked to per line CV in metabolite accumulation with no detectable effect on average metabolite accumulation (Fig. 5). Similarly, there were seven hotspots statistically significantly enriched only in average metabolite accumulation (Fig. 5). The three loci on chromosome I for average metabolite accumulation had the most specific effects on average (M.AV.I.50, −63 and −83; Fig. 5). There were also four loci that were hotspots for both average and per line CV of metabolite accumulation (M.III.51, M.III.64, M.IV.3 and M.IV.72). Thus, the genetics of per line CV and per line average metabolite accumulation can identify sets of genetic loci that include loci specific for one or the other trait. This suggests that stochastic variance of plant metabolism is a heritable genetic trait distinct from that of per line average.

### Neighborhood effects of CV QTLs within the metabolome

Several recent modelling studies had used predictive models of the metabolic grid and suggested that it was possible for stochastic noise within the metabolome to be constrained to specific regions of the grid [32], [33]. To test if our empirical data shows if the CV QTLs have localized effects on metabolite CV as predicted from the models, we plotted the significant additive effects of each locus within a diagram of the metabolic grid (S4 Fig.). These plots showed that the effects of some QTL on metabolite CV were typically localized to a relatively small region. At the extreme were loci that affected only specific nodes within the detectable primary metabolic grid, such as M.CV.V.97 and M.CV.II.16 (S4 Fig.). In contrast to the predictions, there were a number of loci that had wide ranging effects scattered throughout the metabolic grid, such as M.CV.III.51,M. III.64 and M.CV.IV.72 (S4 Fig.). These effects were both positive and negative within the same metabolome. For example, M.CV.III.51 showed increased variance in succinate and xylose while decreased variance in spermidine, glycerate, glu-1-P and other metabolites (S4 Fig.). Thus, in contrast to the modelling studies, it is possible for genetic loci to have wide ranging and opposing effects upon metabolome stochastic variance.

### Mapping QTL for growth and defense chemistry CV

**Figure 6:**
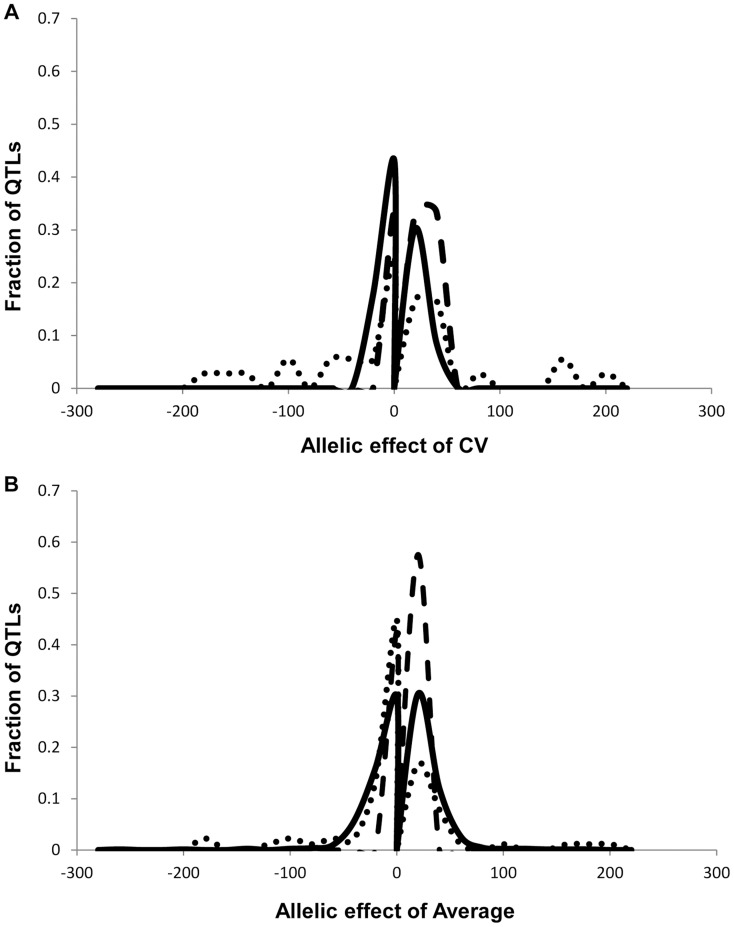
Comparison of estimated additive effects across phenotype classes. The distribution of percent additive effects for nuclear loci is shown for the metabolite, defensive and growth phenotypes. Solid lines for metabolites, dotted lines for glucosinolates and dashed lines for growth. A. Shows the distribution of QTL effect sizes for the CV phenotype. B. Shows the distribution of QTL effect sizes for the average phenotype.

To compare how per line CV loci differ across phenotypic classes, we next used per line CV and average for each RIL for growth and defense chemistry to map QTLs for these phenotypes. As for metabolites, this showed that the average phenotype found more QTLs for all traits than that found for per line CV (4, 7 and 7 versus 2, 1 and 1 for aliphatic glucosinolates, indolic glucosinolates and growth respectively) (Fig. 3 and Tables S4 and S5). In contrast to the rest of the metabolome and transcriptome, the effect size of defense chemistry per line CV QTLs was larger than that for the QTLs affecting the average. The CV QTLs have a mean effect of 57 and 42% for aliphatic and indolic glucosinolates, in contrast to the average QTLs having a 40 and 20% effect respectively (Fig. 6 and S3 Table)[51], [56]. Similarly, effect of the per line CV QTLs for growth was also higher than that for average growth, 21% effect versus 10% (S3 Table)[51], [56]. In all growth and defense phenotypes, the distribution of effect sizes for the phenotypic per line CV was statistically higher than for the phenotypic average (t-test, P<0.01). It should be noted that all growth, defense, and metabolite phenotypes were measured on the same plants indicating that these differences are not likely due to different environments or conditions [51], [56]. This increased effect size of QTLs for per line CV of growth and defense chemistry in comparison to that found for the metabolome suggests that the underlying genetics controlling the per line CV of growth and defense chemistry is structured differently between the traits.

A comparison of QTL maps for the defense traits showed that the previously identified and validated GSL.AOP and GSL.Elong loci control both mean and per line CV for the aliphatic glucosinolate (S5 Fig.) [24]. The stochastic variation and mean accumulation of the aliphatic glucosinolates is controlled by the presence or absence of specific enzyme encoding genes in these loci that lead to pleiotropic effects on the glucosinolate regulatory network [24], [60]–[63]. The GSL.AOP and Elong loci were also linked to suggestive hotspots (P<0.1) in the average metabolome with no signature in the metabolome per line CV (Fig. 5). For aliphatic glucosinolates, there is also a per line CV hotspot near the previously validated MYB28 locus, a transcription factor, that also controls the glucosinolate regulatory network to affect stochastic variation of the pathway (S5 Fig.)[24], [64]–[67]. In contrast to the CV analysis of the metabolome, there were no significant hotspots that were unique to defense chemistry per line CV (S5 Fig.).

Mapping per line CV of growth in comparison to average growth identified a number of average QTLs and only two CV loci for growth (Figs. 5 and S5). The growth QTL, GR.I.19 was associated with variation in both average and per line CV of growth while the QTL, GR.III.2, was specific to per line CV in growth (Tables S4 and S5). There were no hotspot in the metabolome or defense chemistry data for the GR.I.19 or the GR.III.2 loci suggesting that the effect of these loci on the altered per line CV in growth was not having a detectable impact on metabolism Interestingly, only one average or per line CV growth locus (GR.IV.2 vs M.IV.3) overlapped with any metabolomics locus in the entire analysis suggesting we identified different genetic loci for the two traits. Together, this shows that we can map loci for per line CV of growth, metabolism, and defense chemistry and identify loci specific to each trait. Thus, per line CV loci are genetically distinct for all three traits and not reflective of a global stochastic noise locus.

#### Cytoplasmic genome effects on metabolome CV

**Figure 7:**
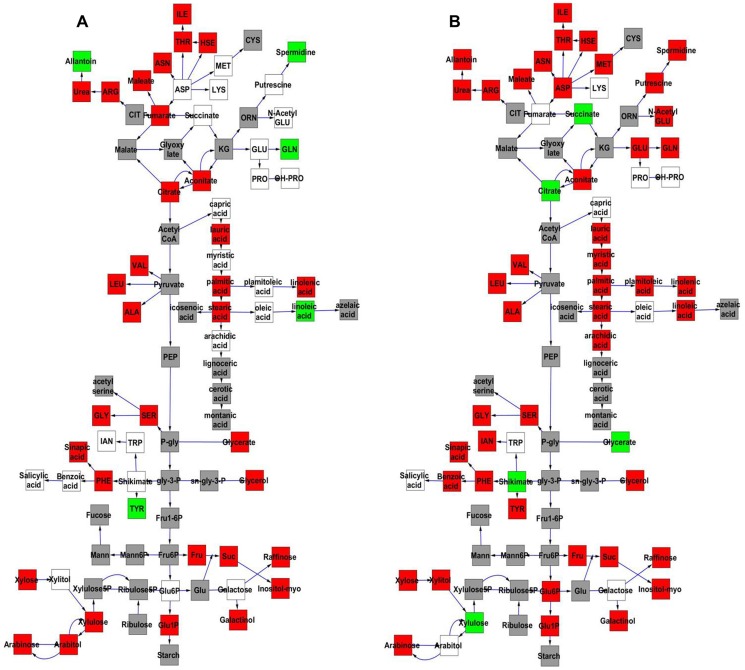
Comparative effect of cytoplasmic genomic variation on Metabolite average and CV. A map of central metabolism was created in cytoscape and used to plot the estimated additive effect of genetic variation in the cytoplasmic genomes. A red box shows increased metabolite accumulation when the line contains the Kas cytoplasmic genome while green shows increased metabolite accumulation when the line contains the Tsu cytoplasmic genome. White boxes are metabolites that were detected but not significantly influenced by the cytoplasmic genome and grey boxes are metabolites that were not detected. A. Metabolites for which the average is significantly affected by the cytoplasmic variation. B. Metabolites for which the CV is affected by the cytoplasmic variation.

The Kas x Tsu population was explicitly established as a reciprocal population with approximately half of the lines having the Kas organellar genomes and the remaining RILs having the Tsu organellar genome. Thus, we explicitly tested if genetic variation within the organellar genomes influenced phenotypic variation in the metabolome, growth, and defense by adding the organellar genome as a term in our single marker linear models (Tables S4 and S5). This analysis showed that genetic variation in the organelle affected variation in per line CV for 422 of the 559 metabolites tested (Tables S4 and S5). The metabolites where per line CV was partly determined by genetic variation in the organelle were spread throughout the metabolic network (Fig. 7). The organellar genome variation affected both the average and per line CV for a subset of metabolites although often with opposite effects (Fig. 7). Organelle genetic variation had opposing effects on average and per line CV for metabolites like tyrosine, glycerate, spermidine, glutamine and citrate (Fig. 7). For example, the Kas organelles lead to lower average glutamine accumulation but higher per line CV of glutamine accumulation (Fig. 7). In addition, there were compounds, like succinate, where the organellar variation affected per line CV but not the average accumulation (Fig. 7). Thus, genomic variation within the chloroplast and/or the mitochondria affects stochastic fluctuations in the steady state metabolome within Arabidopsis.

Intriguingly, 49 of 96 polymorphisms within the mitochondria are found within genes in the NADH complex or in cytochrome C function [51]. These genes are key to controlling NADH metabolism and thus modulating numerous enzymatic reactions within the TCA cycle. Thus, it may not be surprising that two of the metabolites that differed in how the organellar genome influenced average and CV, succinate and citrate, are within the TCA cycle. A more detailed search showed that glycerate, shikimate, and tyrosine are also metabolites whose CV and average are differently affected and their metabolic reactions are also highly dependent on NAD/NADH [68], [69]. Because NADH metabolism provides key cofactors for a large number of metabolic processes, it will require the development of new approaches to manipulate the genes within the organelle to test if these genetic polymorphisms in NADH metabolic genes within the mitochondria can be linked to the differential stochastic variance within the TCA cycle and other metabolic processes.

In contrast to the metabolome, none of the growth traits had either the average or per line CV significantly influenced by the organellar genomic variation. Defense chemistry per line CV also was less affected by the organellar variation with only 5 of 19 phenotypes showing a significant link to organellar genomic variation (Tables S4 and S5). This is similar to the average of these phenotypes where growth and defense were less affected by organellar genomic variation than the metabolome [51], [56]. All metabolome, growth and defense phenotypes were measured on the same plants supporting that the differences in the genetic architecture of per line CV for these traits are not due to differences in the experiment or environment. Thus, the genetic link between the organellar genomes and variation in per line CV of defense chemistry is different than that for the metabolome.

#### Different levels of epistasis for CV and average

**Figure 8:**
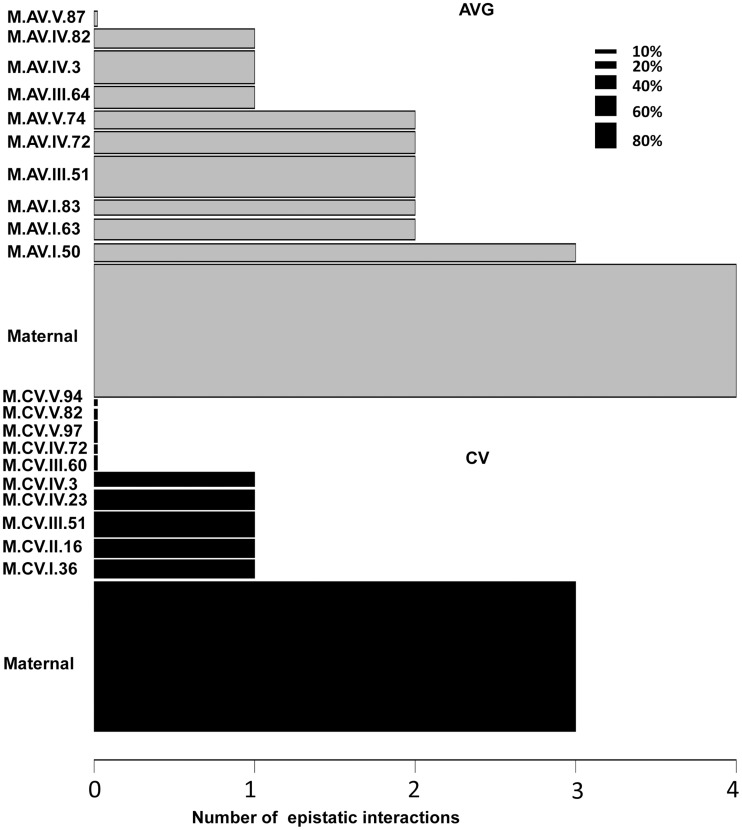
Epistatic interactions of CV and Average QTL hotspots for the metabolome. The bar plots show the number of pairwise epistatic interactions per locus for metabolite CV and Average using the respective QTL hotspots for each phenotype. Grey bars show the analysis with the Average QTL hotspots and black bars represent the CV QTL hot spots. Only the interactions significantly affecting 10% or more metabolites were considered. The width of the bars is scaled to the percentage of metabolites significantly affected by the main effect of that locus.

Using the average values for growth, defense chemistry and the metabolome, we had previously shown that there was extensive epistasis in this population linking the nuclear and organellar genomes [51], [56]. Thus, we tested for epistasis affecting per line CV using a multiple marker model including all hotspots (Tables S6 to S9). There was extensive epistasis for the average metabolite accumulation of these 559 metabolites with each locus having a median of 2 interactions with other loci and only one locus showing no interactions (Fig. 8). This included the organellar genome showing interactions with four different nuclear loci (I.50, III.51, IV.3 and IV.82)(Fig. 8). In contrast, there was significantly less epistasis for per line CV of metabolite accumulation with a median of only 1 interaction per locus and almost half of the loci showing no interactions (Fig. 8). Again, the organellar genome showed the most epistatic interactions and accounted for all detected epistasis involving three nuclear loci for per line CV of metabolite accumulation (I.36, IV.3 and IV.23). Additionally, there were no identifiable three-way epistatic interactions for per line CV of metabolite accumulation, which is in contrast to the average metabolite accumulation where there was extensive multi-locus epistasis [51], [56]. There was also less detectable epistasis for per line CV of growth and defense chemistry in comparison to the average of these traits (S6 and S7 Figs.). This lower fraction of epistasis agrees with the fact that the range of variation across the RILs for per line CV is less than that found for the average of these traits (Fig. 1D). This suggests that the genetic architecture for per line CV of all three trait classes appears to have more additive polygenic basis than that found for the average of these traits (Figs. 1, 5 and 8). In support of this hypothesis, the CV traits are more normally distributed within the RILs than are the averages (S8 Fig.). This is exactly as would be expected for a trait with largely polygenic additive architecture [70]. Alternatively, there could be an unrecognized issue with statistical power in the CV traits in comparison to the mean traits. One possibility is that the median variation of metabolite CV is slightly lower than that for the mean across the RILs (0.48 versus 0.55) across the metabolites. This difference in variation is likely not sufficient to alter the QTL mapping. Another possibility is that the CV may be less normally distributed but an analysis of the distributions showed that CV actually shows more normal distribution across the RILs than does average metabolite accumulation (S8 Fig.). Thus, it appears that this difference in genetic architecture is likely not an issue of the statistical properties of CV in our data. However, further experiments are required to fully validate the hypothesis that CV and average may have a different genetic architecture as has also been suggested for transcripts [24].

## Discussion

Recent work has shown that it was possible to identify genetic loci controlling the stochastic variation in transcript expression within eukaryotes [21], [23], [24]. While modelling analysis suggested that this stochastic variation could permeate into the metabolome, it had been an unresolved question as to how or if there was genetic loci controlling stochastic variation in higher order traits like metabolite accumulation or growth [32], [33]. Using a replicated metabolome and growth analysis of *Arabidopsis thaliana* RILs, we mapped genetic loci controlling stochastic variance in the metabolome, defense chemistry and growth of a multi-cellular eukaryote (Figs. 4 and S2). For all traits, it was possible to find genetic loci that control within line stochastic variance. Additionally for growth and the metabolome, there were loci that specifically affect the stochastic variance with no statistically identifiable effect on the phenotypic average (Figs. 4 and S2). Because all traits were measured on the same individuals, we have minimized any potential for these results to be caused by experimental or environmental variation across the individuals. Thus, it is possible to find genetic loci controlling stochastic variation of traits from transcripts to metabolites to growth in multi-cellular eukaryotes using standard mapping populations and standard replicated experimental design. In agreement with recent modelling studies, our empirical analysis shows stochastic noise can be localized within small neighborhoods of the metabolic network without spreading throughout the system [32], [33]. Further work will be required to map and clone the loci identified to control the stochastic variation in the metabolome and growth.

### Intrinsic stochasticity versus variable plasticity in the face of micro-environmental perturbations

Studies on stochastic variation have difficulty discerning if the observed genetic effects on CV are truly via intrinsic processes. An alternative is that the loci could be reflecting genetic variation that alters the phenotypic plasticity in the presence of micro-environmental perturbations. We would argue that our data is more reflective of intrinsic stochastic variation for the following reasons. First, our experiment was conducted with complete randomization at all levels that should prevent any signature of local environmental structure in technical or biological replicates. Essentially all samples should be equally randomized across any micro-environmental variation. In support of this, diurnally responsive metabolites show all ranges of CV indicating that any effect of micro-diurnal variation on the sampling and CV estimation is minimal (Fig. 2)[71]. Further supporting this is the observation that stress responsive metabolites are not showing elevated CV as would be expected if we were measuring plasticity in response to micro-environmental variation in stress. Secondly, the primary metabolites, secondary metabolites and growth were all measured on the same plants and as such should be exposed to the same micro-environmental variation. Yet the loci identified and genetic architecture of these traits is fundamentally different suggesting that we have mapped loci specific to each metabolic trait and not universal plasticity loci. Thirdly, there were no loci identified with structured global effects in metabolic CV as would be expected if there was the presence of systemic structured biological or technical error (Figs. 7 and S5). Supporting the absence of systemic sources of error came from randomizing the metabolomic data while maintaining the inherent structure. This analysis found that the maximal number of QTLs found was 53 which is only 9% of the 595 CV QTLs identified with the real data arguing against systematic error. Finally, we have previously used this same experimental set up to identify and validate that ELF3 specifically affects intrinsic stochastic noise [24]. Thus, we would argue that while some of our loci may be loci affecting plasticity to extrinsic variance, we have likely identified a number of loci that affect intrinsic stochastic variance within the metabolome and growth in a multi-cellular eukaryote. It will require vastly larger validation experiments to separate which loci are associated with intrinsic vs extrinsic stochastic variance.

### Growth and whole organism stochastic variation

The link between genetic variation and differential stochastic noise in a phenotype has been predominantly studied in single celled organisms [16]–[20], [72]. Additionally, in plants there are whole plant processes that rely on stochastic cell autonomous processes, such as flowering time [73], [74]. This has generated some confusion over the potential for stochastic variation at the whole plant versus cell autonomous level. However, previous work showed that it was possible to identify whole plant stochastic events controlled by genetic polymorphisms buffered by HSP90 [13], [14], [75]. Within our analysis we mapped genetic variation that influenced the stochastic variation of plant growth as measured by the size of the whole rosette. Plant growth is a classical integrative higher-order phenotype like crop yield or disease susceptibility having complex underlying genetics [76], [77]. Thus, it is possible to identify genetic loci that determine the level of stochastic variation in whole plant phenotypes. It remains to be seen if the underlying molecular mechanisms work in cell non-autonomous manners to control whole plant phenotypes or function as stochastic switches in cell autonomous manners that sum up to a whole plant result.

### Organellar variation and stochastic variation

Recent research is beginning to unveil the role of genetic variation within organellar genomes in influencing variation for a range of phenotypes from average metabolite accumulation to growth [51], [56]. Further, only diversity in nuclear encoded genes like *ELF3* have been linked to influencing stochastic variation within plants [24]. Thus, there has not yet been an identified link of the organellar genome variation to controlling different stochastic variation within any organism. Within our study, we found that genomic variation within the organelles lead to a significant impact on the stochastic variation of metabolites as measured by per line CV (Fig. 7). There was also a lesser impact on the defense metabolites and growth (S6 and S7 Figs.). The variation within the organellar genome influenced stochastic variation of primary metabolism differently than average metabolite accumulation. Thus, the organelle genome influences stochastic variation at all phenotypic levels and the CV effects can be separated from the effects on the average phenotypes and these effects are due to genes within the organellar genomes.

### Defense chemistry and stochastic variation

It has been hypothesized that defense related phenotypes benefit from having elevated levels of stochastic variation that generate a bet-hedging-like mechanism whereby a single genotype samples a wider phenotypic range. This can then lead to increases in evolutionary stability of the defense mechanism. Within this experiment, defense metabolites had numerous lines of evidence indicating that they had a higher per line CV and more genetic variation in per line CV than is found for primary metabolites in agreement with this theory. First, defense metabolites have a wider population level variance of per line CV than that found for the other metabolites (aliphatic glucosinolates 1.5±0.3, indolic glucosinolates 0.8±0.3 and primary metabolites 0.5±0.1 [average ± S.E. of population CV for per line CV])(S2 Table). Additionally, we identified more per line CV QTLs for each defense metabolite than for the other metabolites (Fig. 5). Finally, for each identified QTL controlling per line CV, the mean effect for defense metabolites was twice as large as that found for the other metabolites (57% effect for aliphatic glucosinolates, 42% effect for indolic glucosinolates and 22% effect for primary metabolites)(Fig. 6). Taken together, there is a higher level of genetically programmed stochastic variance in glucosinolate defense metabolites in comparison to primary metabolites. Thus, the genetic networks and natural variation influencing defense metabolism in Arabidopsis may be structured to enable higher levels of stochastic variation possibly to mediate bet-hedging interactions within the environment [28], [29].

### Future potential

Within this study, we show that it is possible to identify genetic loci in both the nuclear and organelles that lead to altered stochastic variation in all measured phenotypes from individual metabolites to whole plant growth. Further, these loci differ from trait to trait, suggesting that we are not identifying generic variance loci as might be expected if they were affecting global mechanisms like HSP90. Instead, these CV loci affect specific genetic networks that are distinct for each trait. This suggests that there may be stochastic specific loci for each plant trait. For instance, numerous natural and induced mutant screens and surveys have been conducted in Arabidopsis to determine the genes controlling the phenotypic average [78]–[80]. Similar large scale approaches have been conducted in numerous other organisms focused on phenotypic averages [31], [81], [82]. While these have provided great advances in our understanding of biology, it raises the question of what would happen if we repeat these screens and surveys to identify genetic variation controlling stochastic noise in phenotypes. Would we identify the same genes or would we begin to identify a large suite of previously unknown genes that control stochastic variation rather than phenotypic average? This indicates there is a need for additional experiments focused on stochastic variation within multi-cellular organisms to explore a new avenue of organismal biology.

## Materials and Methods

### Measuring metabolite and growth CV

To directly estimate the CV for each individual metabolites accumulation as a separate phenotype within the Kas x Tsu RIL population [51], [56], we utilized two independent metabolomics experiments in which 316 lines had been measured in duplicate within each experiment [51], [56]. Within each experiment, the 316 lines were planted in randomized complete blocks and all blocks within all experiments were independently randomized. This greatly diminishes any potential for correlated errors in the analysis. Additionally, the metabolomics samples were also randomized prior to injection within the block structure. Again all randomization was independent across blocks for the metabolomics. Only 559 metabolites were measured in all four samples of the previous experiment and we focused solely on these signals to maximize our power to measure metabolite CV [51], [56]. To measure growth and defense compound CV, we obtained the raw data where the plants had also been measured for daily growth (5 growth phenotypes) and glucosinolate accumulation (19 glucosinolate phenotypes) [51], [56]. For each phenotype, metabolite and growth, we utilized the absolute phenotypic values to measure the CV for each phenotype separately for each experiment using σ/µ [16], [21], [83], thus providing two independent biological replicate measures of CV for each phenotype. The use of CV as a direct phenotype has previously been used in a number of instances. By measuring the within line CV as a phenotype for the Kas x Tsu population allows us to then utilize CV as a direct measurement of stochastic variation as a phenotype. The level of per line replication for the array data does not support the use of Levene's variance tests or measures. Additionally, all lines were planted and harvested within a randomized complete block design at all stages thus limiting any potential technical bias to generate these observations [41], [84]. Similarly, the metabolomics analysis was conducted with mixed internal standards run approximately every 20 samples to normalize all of the runs to minimize any potential technical error from the instrument [85]–[87].

### Estimation of CV heritability

For estimating broad-sense heritability, we utilized the independent measures of CV directly as a phenotypic measure. All RIL lines were represented in every block in both experiments creating a perfectly balanced randomized complete block design. All phenotypic data was used to calculate estimates of broad-sense heritability (H) for each phenotype as H =  σ^2^_g_/σ^2^_p_, where σ^2^_g_ was estimated for both the RIL genotypes and cytoplasmic genotypes and σ^2^_p_ was the total phenotypic variance for a trait [88]. The ANOVA model (Line heritability Model) for each metabolite phenotype in each line (y_gmeb_) was: 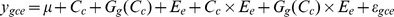 where *c* =  the Kas or Tsu cytoplasm; *g* =  the 1…316 for the 316 RILs, e =  experiment 1 or 2. This allowed cytoplasmic effects to be directly tested in the C term and each RIL genotype (G) nested within the appropriate cytoplasmic class, either Kas or Tsu. Experiment was treated as a random term within the model to better parse the variation. All resulting variance estimates, P-values and heritability terms are presented (S1 Table). σ^2^_g_ for RIL was pulled from the *G_g_(C _c_)* term while σ^2^_g_ for cytoplasmic variation was pulled directly from the *C _c_M_m_* term. We used mean CV values for each RIL for further analysis as we had a randomized complete block design with no missing lines (S2 Table).

### QTL analysis

We used the previously reported genetic map for these lines of the Kas × Tsu RIL population [56], [57]. To detect CV QTLs, we used the average CV per phenotype per RIL across all experiments (S2 Table)[56], [57]. For QTL detection, composite interval mapping (CIM) was implemented using cim function in R/qtl package with a 10 cM window. Forward regression was used to identify three cofactors per trait. To control for genome-wide false positive rates, declaration of statistically significant QTLs was based on permutation-derived empirical thresholds using 1,000 permutations for each mapped trait which yielded a range of LOD significances of 1.8–3.5 to call significant QTLs. In addition to setting a significance threshold, this approach also randomizes the genotype-to-phenotype link to establish a false positive rate. To be conservative, QTLs with a LOD score above 2 were considered significant for further analysis [89], [90]. Composite interval mapping to assign significance based on the underlying trait distribution is robust at handling normal or near normal trait distributions [91], as found for most of our phenotypes. The define peak function implemented in R/eqtl package was used to identify the peak location and one-LOD interval of each significant QTL for each trait [92]. The effectscan function in R/qtl package was used to estimate the QTL additive effect [93]. Allelic effects for each significant QTL are presented as percent effect, by estimating 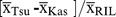 for each significant main effect marker (S3 Table).

QTL clusters were identified using a QTL summation approach where the position of each QTL for each trait was plotted on the chromosome by placing a 1 at the peak of the QTL. This was then used to sum the number of traits that had a detected QTL at a given position using a 5cM sliding window across the genome [94]. The QTL clusters identified defined genetic positions that were named respective to their phenotypic class and genetic positions with a prefix indicating the phenotype followed by the chromosome number and the cM position. For example, M.CV.II.16 indicates a CV metabolomics QTL hotspot on chromosome II at 16 cM. The cluster analysis was conducted separately for metabolomic, defense chemistry and growth phenotypes.

To further assess the potential of structured technical or biological variation to influence our analysis, we conducted a permutation analysis wherein we randomized the line to metabolome links within each of the four randomized blocks. This maintains any correlative structure between the metabolites within a metabolomic sample that may have been caused by structured technical or biological error. We then recalculated CV and mean within each RIL using the randomized phenotype data and used this to re-conduct the entire QTL analysis as described above. 100 permutations of the entire dataset identified a maximum of only 53 metabolomic CV QTL identified across the 559 metabolites in any given permutation which lead to no hotspots being identified. This suggests that the observed hotspots are not caused by structured error within the metabolomics samples.

### Additive ANOVA model

To directly test the additive effect of each identified QTL cluster, we used an ANOVA model containing the markers most closely associated with each of the significant QTL clusters as individual main effect terms. For each metabolite the average accumulation in lines of genotype *g* at marker *m* was shown as *y_gm_*. The model (Additive Model) for each metabolite in each line (y_gm_) was: 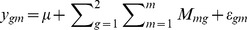


where *g* =  Kas(1) or Tsu(2); *m* = 1, …,11. The main effect of the markers was denoted as *M* involving 15 markers (m). The cytoplasmic genome was included as an additional marker to test for cytoplasmic genome effects. We independently tested the average metabolite accumulation and CV of each metabolite as a separate phenotype with the appropriate model using lm function implemented in the R/car package, which returned all P values, Type III sums-of-squares for the complete model and each main effect. The results using the average metabolite accumulation are presented (S4 Table) separately from those for the CV of metabolite accumulation (S5 Table). QTL main-effect estimates (in terms of allelic substitution values) were estimated for each marker [93], [95]. The same analysis was conducted for the aliphatic glucosinolates, indolic glucosinolates and growth except that these phenotypes only had 9 loci instead of 10 (Tables S4 and S5). There is no significant single marker or pairwise segregation distortion in this population indicating that the model is balanced for all markers [57].

### QTL epistasis analysis

To test directly for epistatic interactions between the detected QTLs, we conducted an ANOVA using the pairwise epistasis model. We used this pairwise epistasis model per metabolite because we had previous evidence that RIL populations have a significant false negative QTL detection issue and wanted to be inclusive of all possible significant loci [49]. Within the model, we tested all possible pairwise interactions between the markers. For each phenotype, the average value in the RILs of genotype *g* at marker *m* was shown as *y_gm_*. The model (Pairwise epistasis model) for each metabolite in each line (y_gm_) was: 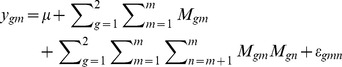



where *g* =  Kas(1) or Tsu(2); *m* = 1, …,14 and n was the identity of the second marker for an interaction. The main effect of the markers was denoted as *M* having a model involving 15 markers. The cytoplasmic genome was included as an additional single-locus marker to test for interactions between the cytoplasmic and nuclear genomes. We independently tested the average metabolite accumulation and CV of each metabolite as a separate phenotype with the appropriate model using lm function implemented in the R/car package, which returned all P values, Type III sums-of-squares for the complete model and each main effect. The results using the average metabolite accumulation are presented (Tables S6 and S7) separately from those for the CV of metabolite accumulation (Tables S8 and S9). Significance values were corrected for multiple testing within a model using FDR (<0.05). The main effect and epistatic interactions of the loci were visualized using cytoscape.v2.8.3 with interactions significant for less than 10% of the phenotypes were excluded from the network analysis [44], [96]. The 10% threshold was chosen as an additional correction for multiple testing to provide a more conservative image of the network. The same analysis was conducted for the aliphatic glucosinolates, indolic glucosinolates and growth except that these phenotypes only had 9 loci instead of 10 (Tables S6 to S9). There are no pairwise locus segregation distortions within this population showing that the genotypes in this analysis are balanced [57].

## Supporting Information

S1 Fig Comparison of CV and Average genetics in Kas x Tsu for growth and defense. Comparison of estimated metabolite heritability's using each metabolites CV (black) and average (grey) phenotype across Kas x Tsu RIL populations. A frequency plot shows the estimated heritability's ascribed to the nuclear (solid lines) and organellar (dotted) genomes across all the metabolites. A. Aliphatic Glucosinolate phenotypes. B. Indolic Glucosinolate phenotypes. C. Growth phenotypes.
(TIF)

S2 Fig Relationship between metabolite average and CV across the RILs. Shown is a hexbin plot of the relationship between the mean and CV of each metabolite in each RIL across the entire dataset. The resolution of the plot is set to 50 bins.
(TIF)

S3 Fig Lack of correlation in QTL number and effect for CV and Mean metabolite accumulation. A. Shown is the number of QTLs for a given metabolite for both CV and mean. The size of the pie's is proportionate to the number of metabolites present in that specific grouping. No significant correlation was found using either spearman or pearson tests. B. For metabolites where the CV and mean QTLs 1 LOD interval overlapped, the estimated additive effect on CV and mean are plotted. No significant correlation was found using either spearman or pearson tests.
(TIF)

S4 Fig Effect of CV hotspots on CV across the metabolomic network. A map of central metabolism was created in cytoscape and used to plot the estimated additive effect of genetic variation of each metabolite CV hotspot on the affected primary metabolites. A red box shows increased metabolite accumulation when the line contains the Kas cytoplasmic genome while green shows increased metabolite accumulation when the line contains the Tsu cytoplasmic genome. White boxes are metabolites that were detected but not significantly influenced by the cytoplasmic genome and grey boxes are metabolites that were not detected. Each page represents a unique metabolite CV hotspot.
(PDF)

S5 Fig QTL hotspots for defense and growth average and CV. The number of metabolites for which a QTL was detected within a 5 cM sliding window is plotted against the genetic location of the metabolite QTLs in cM. Metabolite average QTLs are shown in grey and CV in black. A. Aliphatic Glucosinolate phenotypes. B. Indolic Glucosinolate phenotypes. C. Growth phenotypes.
(TIF)

S6 Fig Epistatic interactions of Glucosinolate phenotypes using both the average and CV QTL hotspots. The bar plots show the number of pairwise epistatic interactions per locus for Aliphatic (A) and Indole (B) glucosinolate CV and Average using the respective QTL hotspots for each phenotype. Grey bars show the analysis with the Average QTL hotspots and black bars represent the CV QTL hot spots. Only the interactions significantly affecting 10% or more metabolites were considered. The width of the bars is scaled to the percentage of metabolites significantly affected by the main effect of that locus as shown.
(TIF)

S7 Fig Epistatic interactions of growth phenotypes using both the average and CV QTL hotspots. The bar plots show the number of pairwise epistatic interactions per locus for growth CV and Average using the respective QTL hotspots for each phenotype. Grey bars show the analysis with the Average QTL hotspots and black bars represent the CV QTL hot spots. Only the interactions significantly affecting 10% or more metabolites were considered. The width of the bars is scaled to the percentage of metabolites significantly affected by the main effect of that locus as shown.
(TIF)

S8 Fig Differential normality of CV and Mean across the RILs. For each metabolite, the skewness and kurtosis was measured for both CV and mean across the RILs. The distribution of these values across the metabolites for both CV and mean(AV) are shown.
(TIF)

S1 Table Estimation of Heritability for Metabolite CV. The results of the linear model analyzing the variation of CV across the Experiments and two estimatible genomes (Organellar and Nuclear) are shown.
(XLSX)

S2 Table Average per line CV for all phenotypes. Shown is the average per line CV for each RIL for each phenotype as estimated from the ANOVA.
(XLSX)

S3 Table QTLs identified for per line CV for all phenotypes. The position and estimated effect size for each identified QTL for each phenotype is presented.
(XLSX)

S4 Table Results of single marker ANOVA model testing QTL effects for average phenotypes. Results of the single marker validation modeling using the QTL hotspots found in this analysis with the average phenotypes. Metabolites are at the start with defense compounds and growth at the bottom of the table.
(XLSX)

S5 Table Results of single marker ANOVA model testing QTL effects for per line CV phenotypes. Results of the single marker validation modeling using the QTL hotspots found in this analysis with the per line CV phenotypes. Metabolites are at the start with defense compounds and growth at the bottom of the table.
(XLSX)

S6 Table P values of Pairwise epistasis tests using ANOVA for all average phenotypes. Results of the pairwise epistasis analysis using the average phenotypes and QTLs validated from the single marker ANOVA. Only P values for the model are shown in this table. Metabolites are at the start with defense compounds and growth at the bottom of the table.
(XLSX)

S7 Table Type III Sums of squares for Pairwise epistasis tests using ANOVA for all average phenotypes. Results of the pairwise epistasis analysis using the average phenotypes and QTLs validated from the single marker ANOVA. Only type III Sums-of-square values for the model are shown in this table. Metabolites are at the start with defense compounds and growth at the bottom of the table.
(XLSX)

S8 Table P values of Pairwise epistasis tests using ANOVA for all per line CV phenotypes. Results of the pairwise epistasis analysis using per line CV phenotypes and QTLs validated from the single marker ANOVA. Only P values for the model are shown in this table. Metabolites are at the start with defense compounds and growth at the bottom of the table.
(XLSX)

S9 Table Type III Sums of squares for Pairwise epistasis tests using ANOVA for all per line CV phenotypes. Results of the pairwise epistasis analysis using per line CV phenotypes and QTLs validated from the single marker ANOVA. Only type III Sums-of-Squares values for the model are shown in this table. Metabolites are at the start with defense compounds and growth at the bottom of the table.
(XLSX)
