# Supplementary figures and images for: Genetic Variation in the Nuclear and Organellar Genomes Modulates Stochastic Variation in the Metabolome, Growth, and Defense
Source: PLoS Genet. 2015 Jan 8;11(1):e1004779. doi: 10.1371/journal.pgen.1004779 (PMC4287608; doi:10.1371/journal.pgen.1004779)

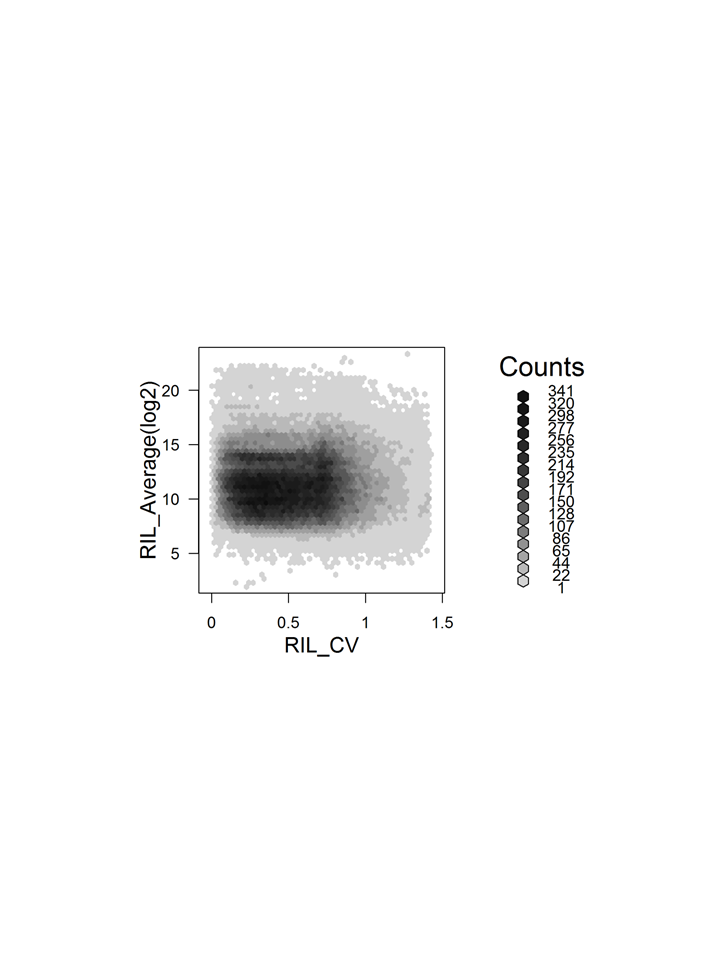

Supplement: S2 Fig — Relationship between metabolite average and CV across the RILs. Shown is a hexbin plot of the relationship between the mean and CV of each metabolite in each RIL across the entire dataset. The resolution of the plot is set to 50 bins. (TIF) [file pgen.1004779.s002.tif]

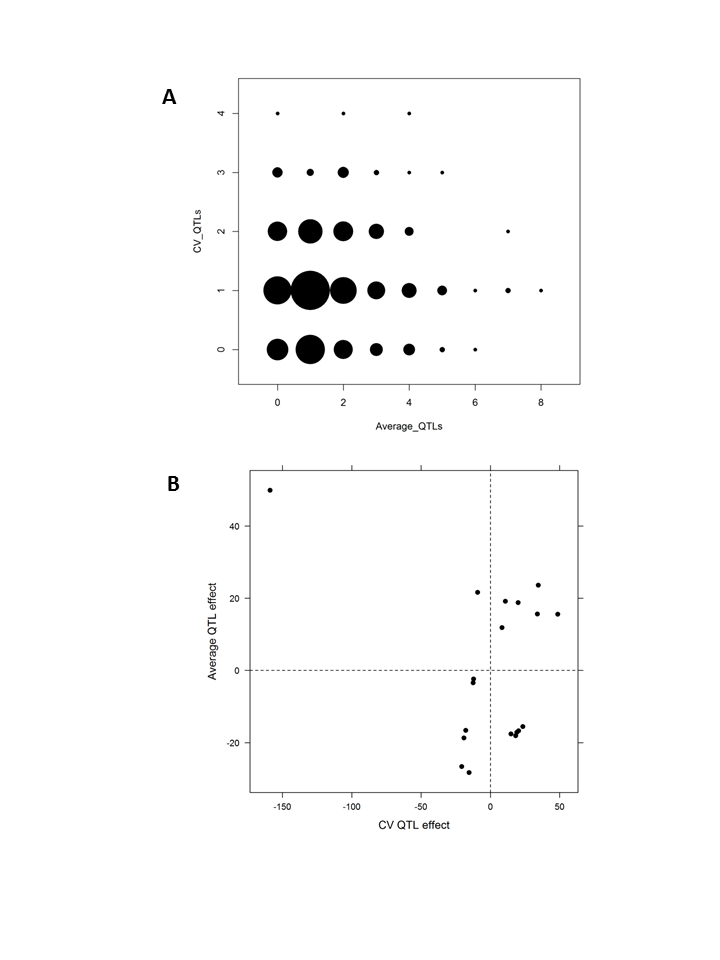

Supplement: S3 Fig — Lack of correlation in QTL number and effect for CV and Mean metabolite accumulation. A. Shown is the number of QTLs for a given metabolite for both CV and mean. The size of the pie's is proportionate to the number of metabolites present in that specific grouping. No significant correlation was found using either spearman or pearson tests. B. For metabolites where the CV and mean QTLs 1 LOD interval overlapped, the estimated additive effect on CV and mean are plotted. No significant correlation was found using either spearman or pearson tests. (TIF) [file pgen.1004779.s003.tif]

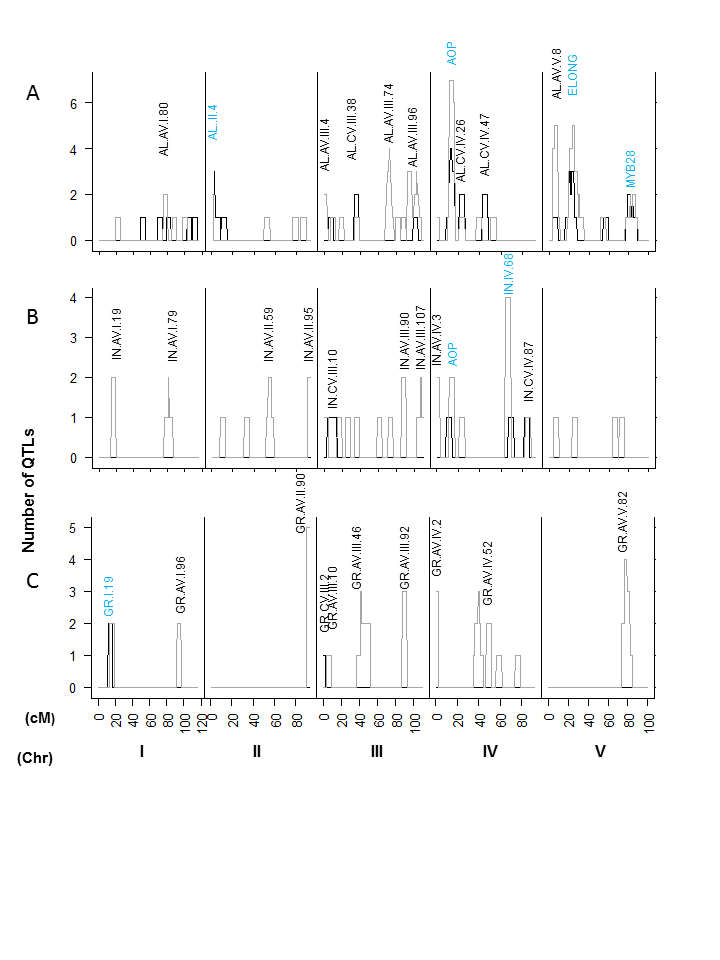

Supplement: S5 Fig — QTL hotspots for defense and growth average and CV. The number of metabolites for which a QTL was detected within a 5 cM sliding window is plotted against the genetic location of the metabolite QTLs in cM. Metabolite average QTLs are shown in grey and CV in black. A. Aliphatic Glucosinolate phenotypes. B. Indolic Glucosinolate phenotypes. C. Growth phenotypes. (TIF) [file pgen.1004779.s005.tif]

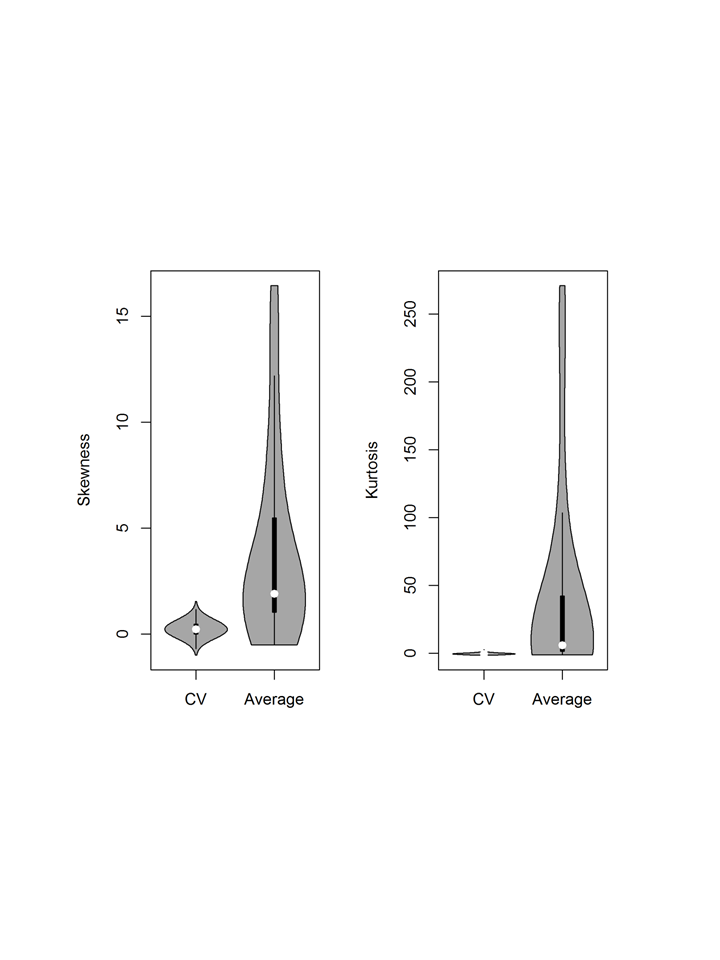

Supplement: S8 Fig — Differential normality of CV and Mean across the RILs. For each metabolite, the skewness and kurtosis was measured for both CV and mean across the RILs. The distribution of these values across the metabolites for both CV and mean(AV) are shown. (TIF) [file pgen.1004779.s008.tif]
