# Supplementary material for: Genetic Variation in the Nuclear and Organellar Genomes Modulates Stochastic Variation in the Metabolome, Growth, and Defense
Source: PLoS Genet. 2015 Jan 8;11(1):e1004779. doi: 10.1371/journal.pgen.1004779 (PMC4287608; doi:10.1371/journal.pgen.1004779)
Supplement: S4 Fig — Effect of CV hotspots on CV across the metabolomic network. A map of central metabolism was created in cytoscape and used to plot the estimated additive effect of genetic variation of each metabolite CV hotspot on the affected primary metabolites. A red box shows increased metabolite accumulation when the line contains the Kas cytoplasmic genome while green shows increased metabolite accumulation when the line contains the Tsu cytoplasmic genome. White boxes are metabolites that were detected but not significantly influenced by the cytoplasmic genome and grey boxes are metabolites that were not detected. Each page represents a unique metabolite CV hotspot. (PDF) [file pgen.1004779.s004.pdf]

M.CV.I.36

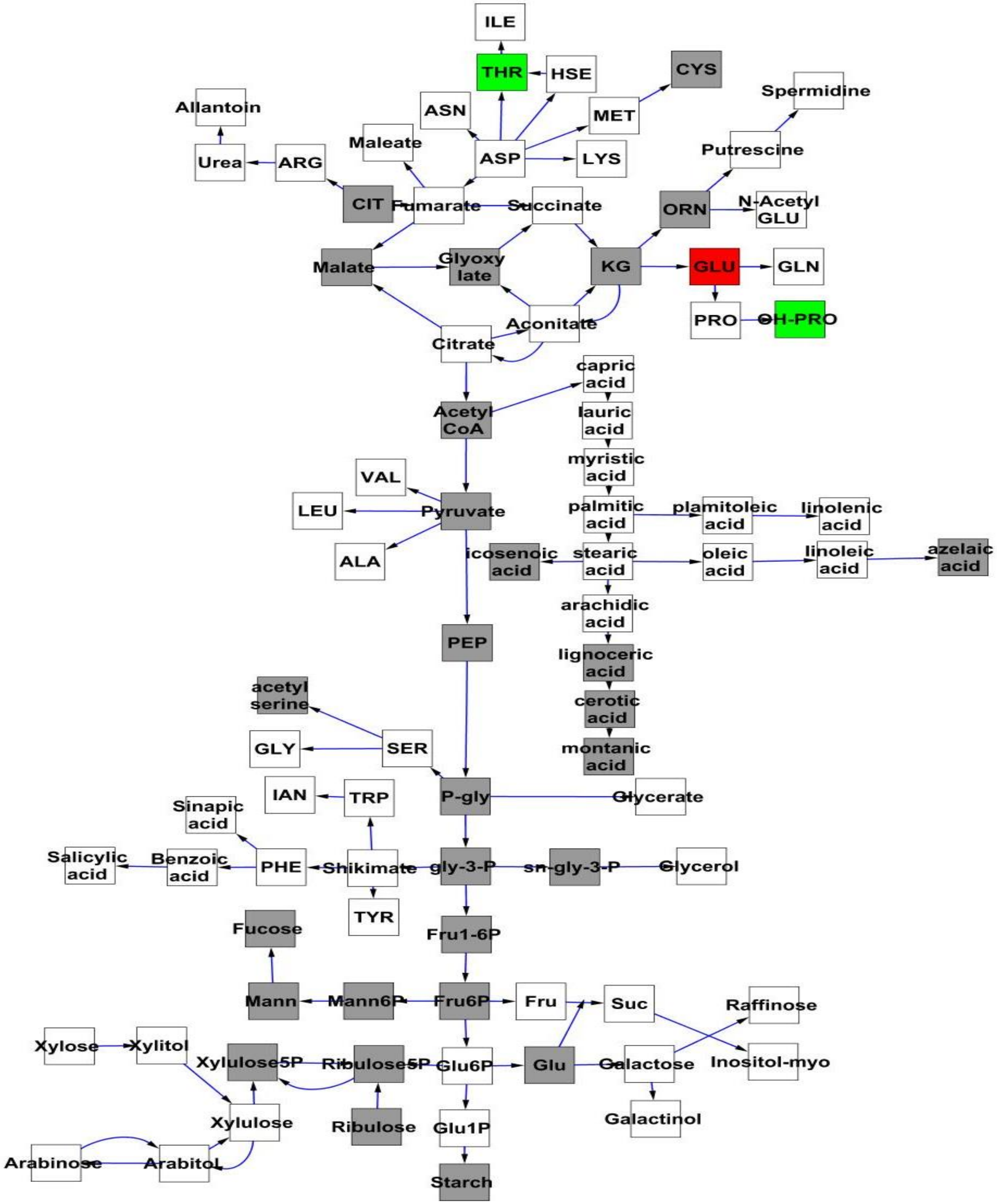
ILE
THR
HSE
CYS
ASN
MET
Spermidine
Allantoin
Maleate
ASP
LYS
Putrescine
Urea
ARG
CIT
Fumarate
Succinate
ORN
N-Acetyl GLU
Malate
Glyoxy late
KG
GLU
GLN
Aconitate
PRO
OH-PRO
Citrate
capric acid
Acetyl CoA
lauric acid
myristic acid
VAL
LEU
Pyruvate
palmitic acid
plamitoleic acid
linolenic acid
ALA
icosenoic acid
stearic acid
oleic acid
linoleic acid
azelaic acid
arachidic acid
PEP
lignoceric acid
acetyl serine
cerotic acid
GLY
SER
montanic acid
IAN
TRP
P-gly
Glycerate
Sinapic acid
Salicylic acid
Benzoic acid
PHE
Shikimate
gly-3-P
sn-gly-3-P
Glycerol
TYR
Fucose
Fru1-6P
Mann
Mann6P
Fru6P
Fru
Suc
Raffinose
Xylose
Xylitol
Xylulose5P
Ribulose5P
Glu6P
Glu
Galactose
Inositol-myo
Xylulose
Ribulose
Glu1P
Galactinol
Arabinose
Arabitol
Starch

M.CV.II.16

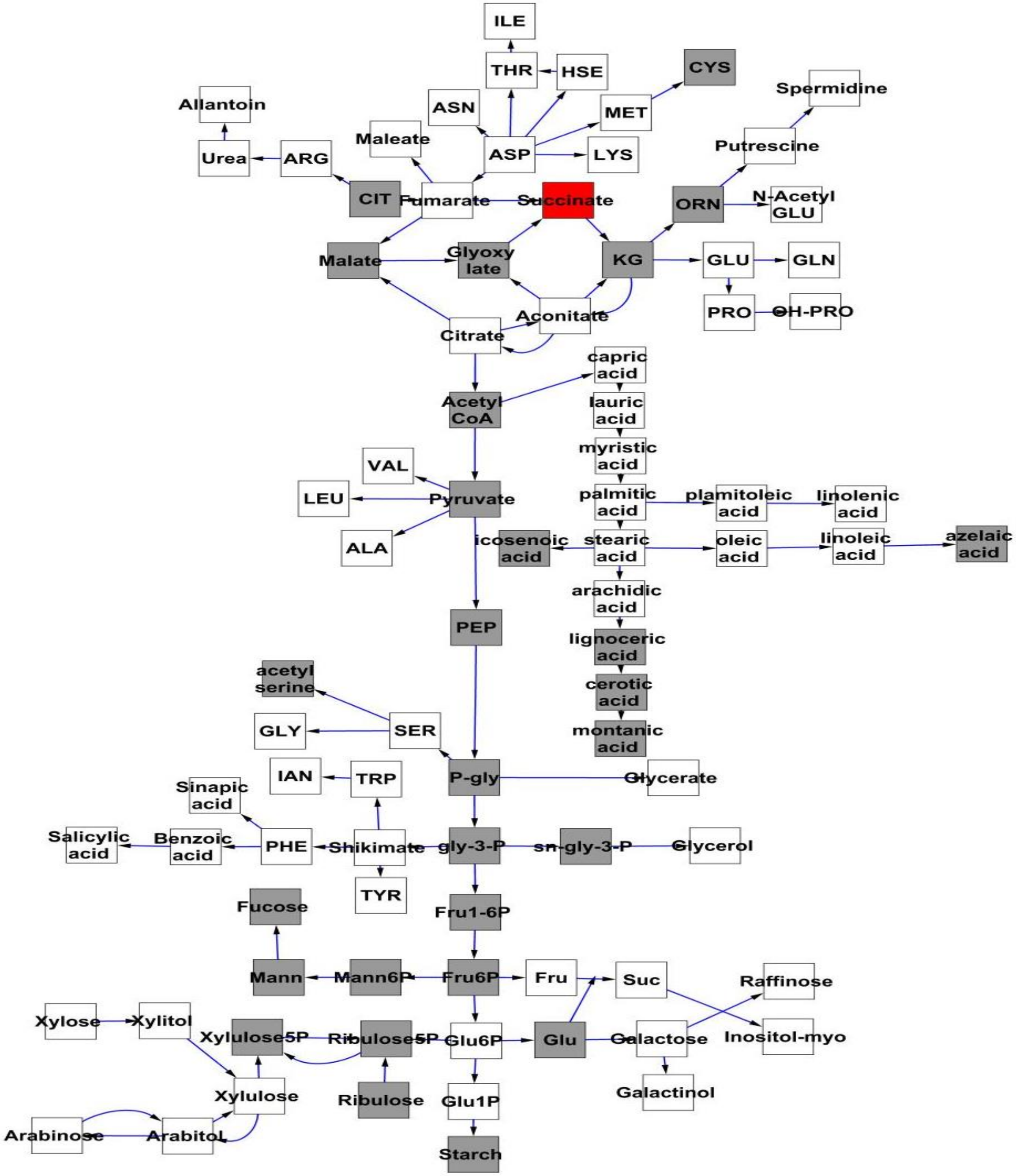

ILE
THR
HSE
CYS
ASN
MET
Spermidine
Allantoin
Maleate
ASP
LYS
Urea
ARG
Putrescine
CIT
Fumarate
Succinate
ORN
N-Acetyl GLU
Malate
Glyoxy late
KG
GLU
GLN
PRO
OH-PRO
Aconitate
Citrate
capric acid
Acetyl CoA
lauric acid
myristic acid
VAL
LEU
Pyruvate
palmitic acid
plamitoleic acid
linolenic acid
ALA
icosenoic acid
stearic acid
oleic acid
linoleic acid
azelaic acid
arachidic acid
PEP
lignoceric acid
acetyl serine
cerotic acid
GLY
SER
montanic acid
IAN
TRP
P-gly
Glycerate
Sinapic acid
Salicylic acid
Benzoic acid
PHE
Shikimate
gly-3-P
sn-gly-3-P
Glycerol
TYR
Fucose
Fru1-6P
Mann
Mann6P
Fru6P
Fru
Suc
Raffinose
Xylose
Xylitol
Xylulose5P
Ribulose5P
Glu6P
Glu
Galactose
Inositol-myo
Xylulose
Ribulose
Glu1P
Galactinol
Arabinose
Arabitol
Starch

M.CV.III.51

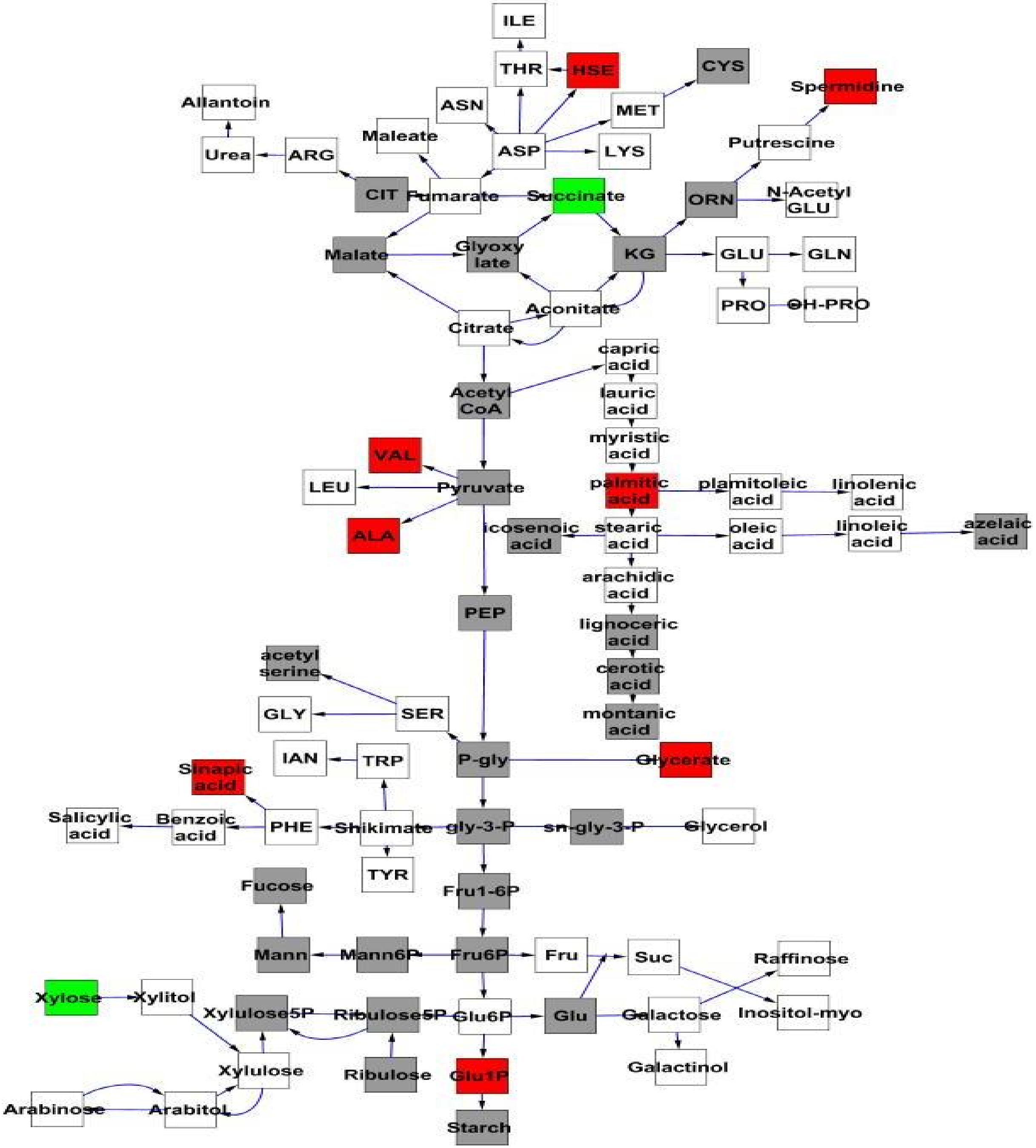

ILE
THR
HSE
CYS
ASN
MET
Spermidine
Allantoin
Maleate
ASP
LYS
Putrescine
Urea
ARG
CIT
Fumarate
Succinate
ORN
N-Acetyl GLU
Malate
Glyoxy late
KG
GLU
GLN
PRO
OH-PRO
Aconitate
Citrate
capric acid
Acetyl CoA
lauric acid
myristic acid
VAL
LEU
Pyruvate
palmitic acid
plamitoleic acid
linolenic acid
ALA
icosenoic acid
stearic acid
oleic acid
linoleic acid
azelaic acid
arachidic acid
PEP
lignoceric acid
acetyl serine
cerotic acid
GLY
SER
montanic acid
IAN
TRP
P-gly
Glycerate
Sinapic acid
Salicylic acid
Benzoic acid
PHE
Shikimate
gly-3-P
sn-gly-3-P
Glycerol
TYR
Fucose
Fru1-6P
Mann
Mann6P
Fru6P
Fru
Suc
Raffinose
Xylose
Xylitol
Xylulose5P
Ribulose5P
Glu6P
Glu
Galactose
Inositol-myo
Xylulose
Ribulose
Glu1P
Galactinol
Arabinose
Arabitol
Starch

M.CV.III.64

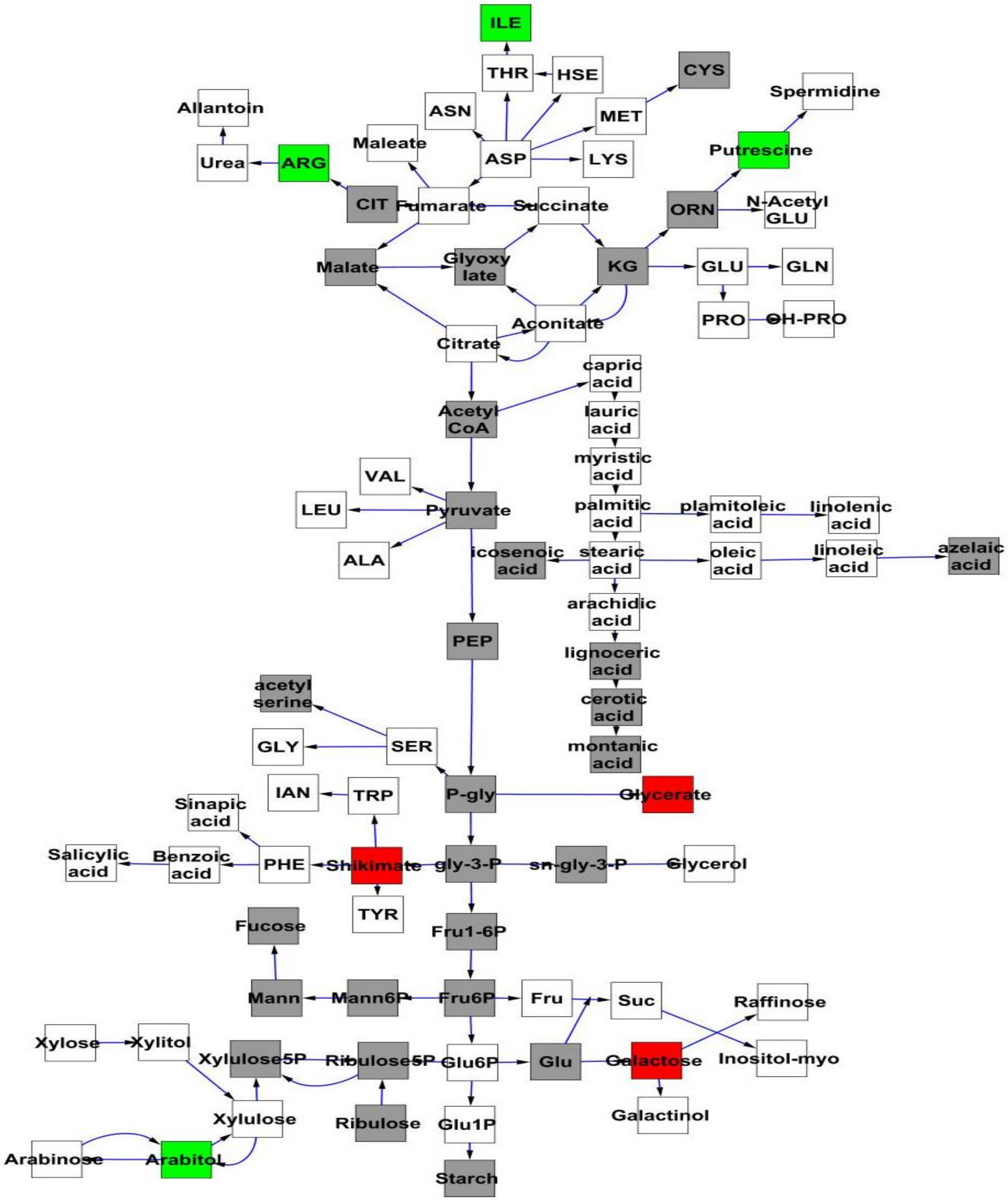

ILE
THR
HSE
CYS
ASN
MET
Spermidine
Allantoin
Maleate
ASP
LYS
Putrescine
Urea
ARG
CIT
Fumarate
Succinate
ORN
N-Acetyl GLU
Malate
Glyoxy late
KG
GLU
GLN
PRO
OH-PRO
Aconitate
Citrate
capric acid
Acetyl CoA
lauric acid
myristic acid
VAL
LEU
Pyruvate
palmitic acid
plamitoleic acid
linolenic acid
ALA
icosenoic acid
stearic acid
oleic acid
linoleic acid
azelaic acid
arachidic acid
PEP
lignoceric acid
acetyl serine
cerotic acid
GLY
SER
montanic acid
IAN
TRP
P-gly
Glycerate
Sinapic acid
Salicylic acid
Benzoic acid
PHE
Shikimate
gly-3-P
sn-gly-3-P
Glycerol
TYR
Fucose
Fru1-6P
Mann
Mann6P
Fru6P
Fru
Suc
Raffinose
Xylose
Xylitol
Xylulose5P
Ribulose5P
Glu6P
Glu
Galactose
Inositol-myo
Xylulose
Ribulose
Glu1P
Galactinol
Arabinose
Arabitol
Starch

M.CV.IV.3

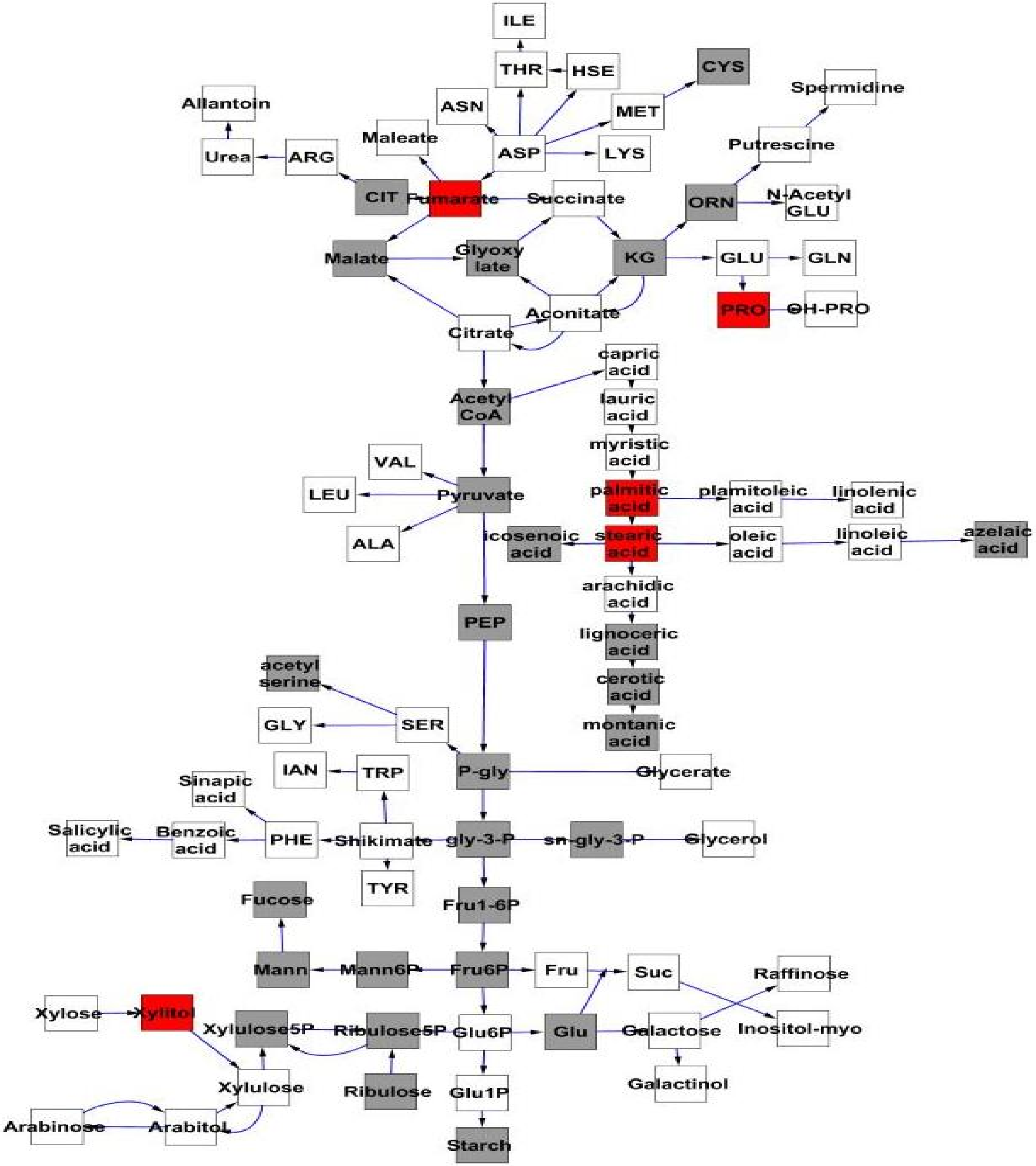
ILE
THR
HSE
CYS
ASN
MET
Spermidine
Allantoin
Maleate
ASP
LYS
Putrescine
Urea
ARG
CIT
Fumarate
Succinate
ORN
N-Acetyl GLU
Malate
Glyoxy late
KG
GLU
GLN
PRO
OH-PRO
Aconitate
Citrate
capric acid
Acetyl CoA
lauric acid
myristic acid
VAL
LEU
Pyruvate
palmitic acid
plamitoleic acid
linolenic acid
ALA
icosenoic acid
stearic acid
oleic acid
linoleic acid
azelaic acid
arachidic acid
PEP
lignoceric acid
acetyl serine
cerotic acid
GLY
SER
montanic acid
IAN
TRP
P-gly
Glycerate
Sinapic acid
Salicylic acid
Benzoic acid
PHE
Shikimate
gly-3-P
sn-gly-3-P
Glycerol
TYR
Fucose
Fru1-6P
Mann
Mann6P
Fru6P
Fru
Suc
Raffinose
Xylose
Xylitol
Xylulose5P
Ribulose5P
Glu6P
Glu
Galactose
Inositol-myo
Galactinol
Xylulose
Ribulose
Glu1P
Arabinose
Arabitol
Starch

M.CV.IV.23

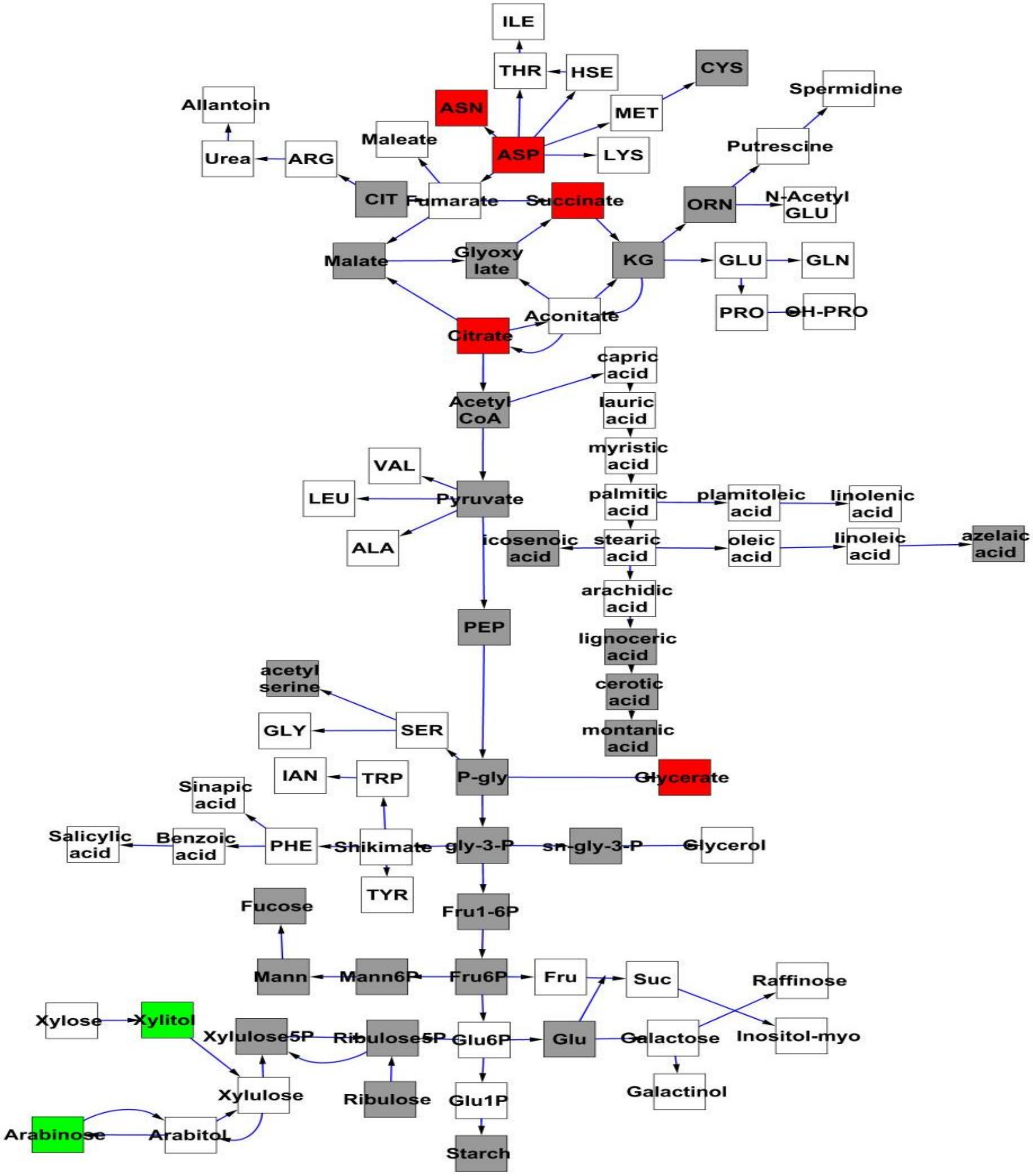
ILE
THR
HSE
CYS
ASN
MET
Spermidine
Allantoin
Maleate
ASP
LYS
Putrescine
Urea
ARG
CIT
Fumarate
Succinate
ORN
N-Acetyl GLU
Malate
Glyoxy late
KG
GLU
GLN
PRO
OH-PRO
Aconitate
Citrate
capric acid
Acetyl CoA
lauric acid
myristic acid
VAL
LEU
Pyruvate
palmitic acid
plamitoleic acid
linolenic acid
ALA
icosenoic acid
stearic acid
oleic acid
linoleic acid
azelaic acid
arachidic acid
PEP
lignoceric acid
acetyl serine
cerotic acid
GLY
SER
montanic acid
IAN
TRP
P-gly
Glycerate
Sinapic acid
Salicylic acid
Benzoic acid
PHE
Shikimate
gly-3-P
sn-gly-3-P
Glycerol
TYR
Fucose
Fru1-6P
Mann
Mann6P
Fru6P
Fru
Suc
Raffinose
Xylose
Xylitol
Xylulose5P
Ribulose5P
Glu6P
Glu
Galactose
Inositol-myo
Xylulose
Ribulose
Glu1P
Galactinol
Arabinose
Arabitol
Starch

M.CV.IV.72

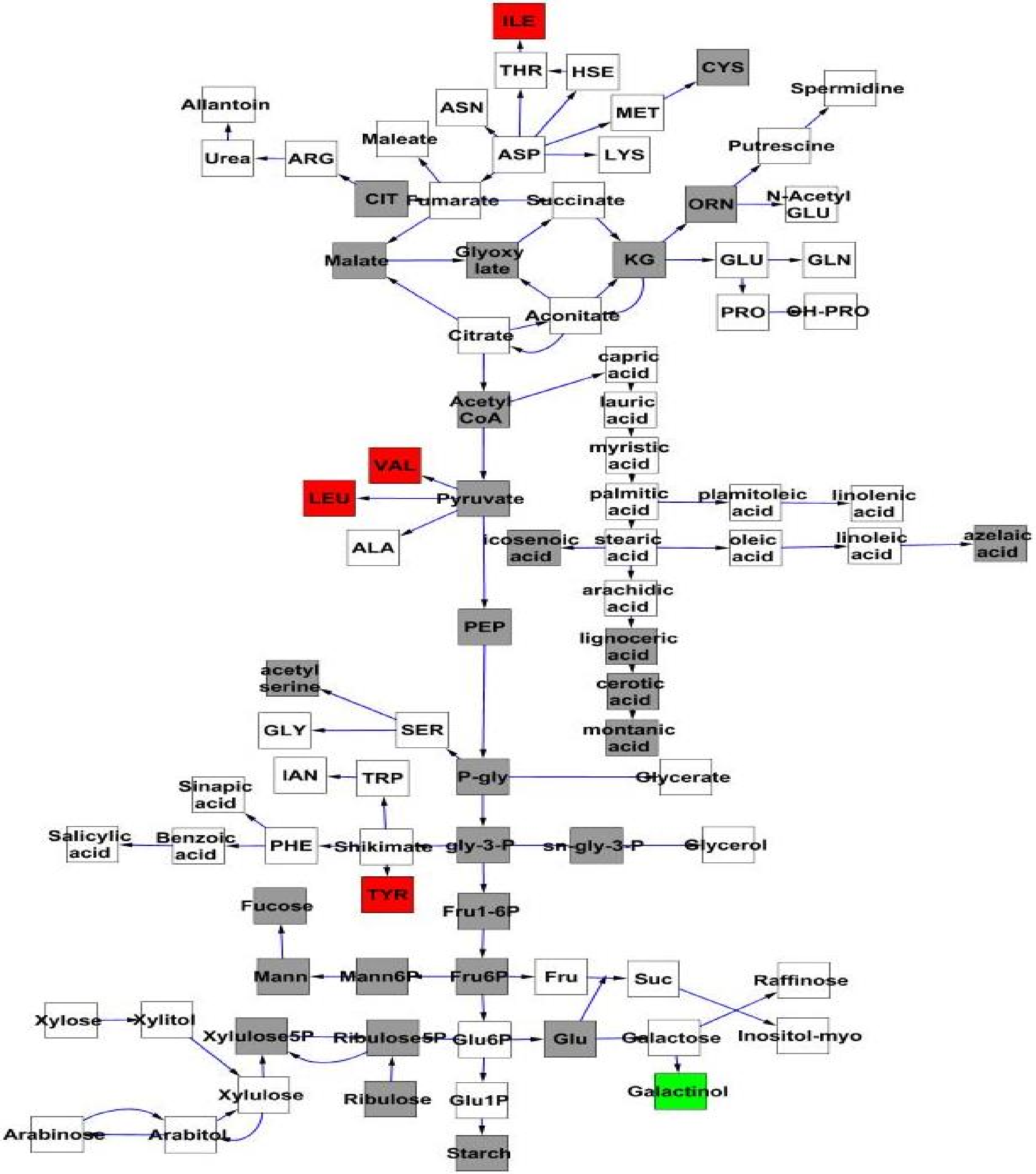
ILE
THR
HSE
CYS
ASN
MET
Spermidine
Allantoin
Maleate
ASP
LYS
Putrescine
Urea
ARG
CIT
Fumarate
Succinate
ORN
N-Acetyl GLU
Malate
Glyoxy late
KG
GLU
GLN
PRO
OH-PRO
Aconitate
Citrate
capric acid
Acetyl CoA
lauric acid
myristic acid
VAL
LEU
Pyruvate
palmitic acid
plamitoleic acid
linolenic acid
ALA
icosenoic acid
stearic acid
oleic acid
linoleic acid
azelaic acid
arachidic acid
PEP
lignoceric acid
acetyl serine
cerotic acid
GLY
SER
montanic acid
IAN
TRP
P-gly
Glycerate
Sinapic acid
Salicylic acid
Benzoic acid
PHE
Shikimate
gly-3-P
sn-gly-3-P
Glycerol
TYR
Fucose
Fru1-6P
Mann
Mann6P
Fru6P
Fru
Suc
Raffinose
Xylose
Xylitol
Xylulose5P
Ribulose5P
Glu6P
Glu
Galactose
Inositol-myo
Xylulose
Ribulose
Glu1P
Galactinol
Arabinose
Arabitol
Starch

M.CV.V.94

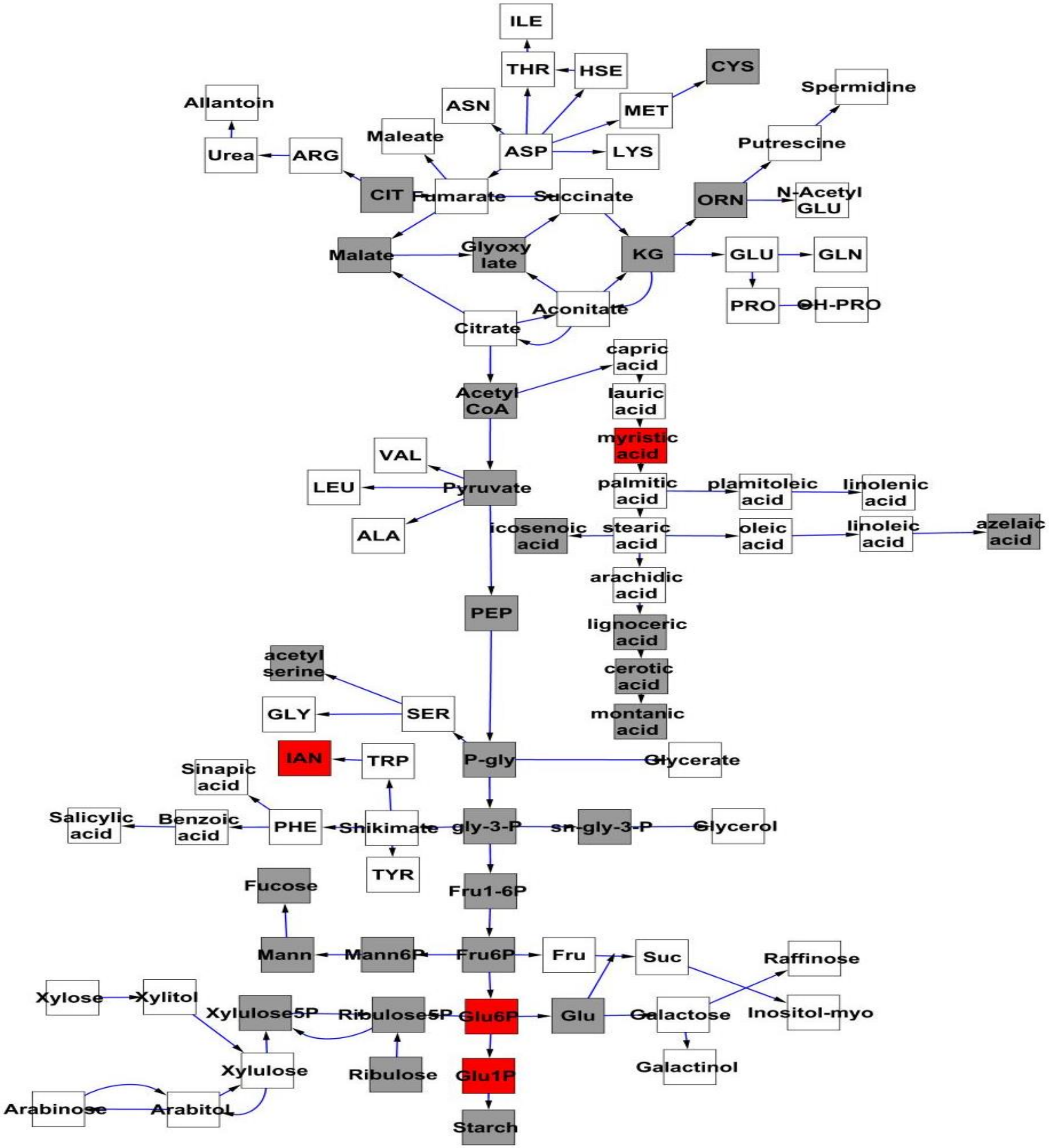
ILE
THR
HSE
CYS
Spermidine
ASN
MET
Allantoin
Maleate
ASP
LYS
Putrescine
Urea
ARG
CIT
Fumarate
Succinate
ORN
N-Acetyl GLU
Malate
Glyoxy late
KG
GLU
GLN
PRO
OH-PRO
Aconitate
Citrate
capric acid
Acetyl CoA
lauric acid
myristic acid
VAL
LEU
Pyruvate
palmitic acid
plamitoleic acid
linolenic acid
ALA
icosenoic acid
stearic acid
oleic acid
linoleic acid
azelaic acid
arachidic acid
PEP
lignoceric acid
acetyl serine
cerotic acid
GLY
SER
montanic acid
IAN
TRP
P-gly
Glycerate
Sinapic acid
Salicylic acid
Benzoic acid
PHE
Shikimate
gly-3-P
sn-gly-3-P
Glycerol
TYR
Fucose
Fru1-6P
Mann
Mann6P
Fru6P
Fru
Suc
Raffinose
Xylose
Xylitol
Xylulose5P
Ribulose5P
Glu6P
Glu
Galactose
Inositol-myo
Xylulose
Ribulose
Glu1P
Galactinol
Arabinose
Arabitol
Starch

M.CV.V.97

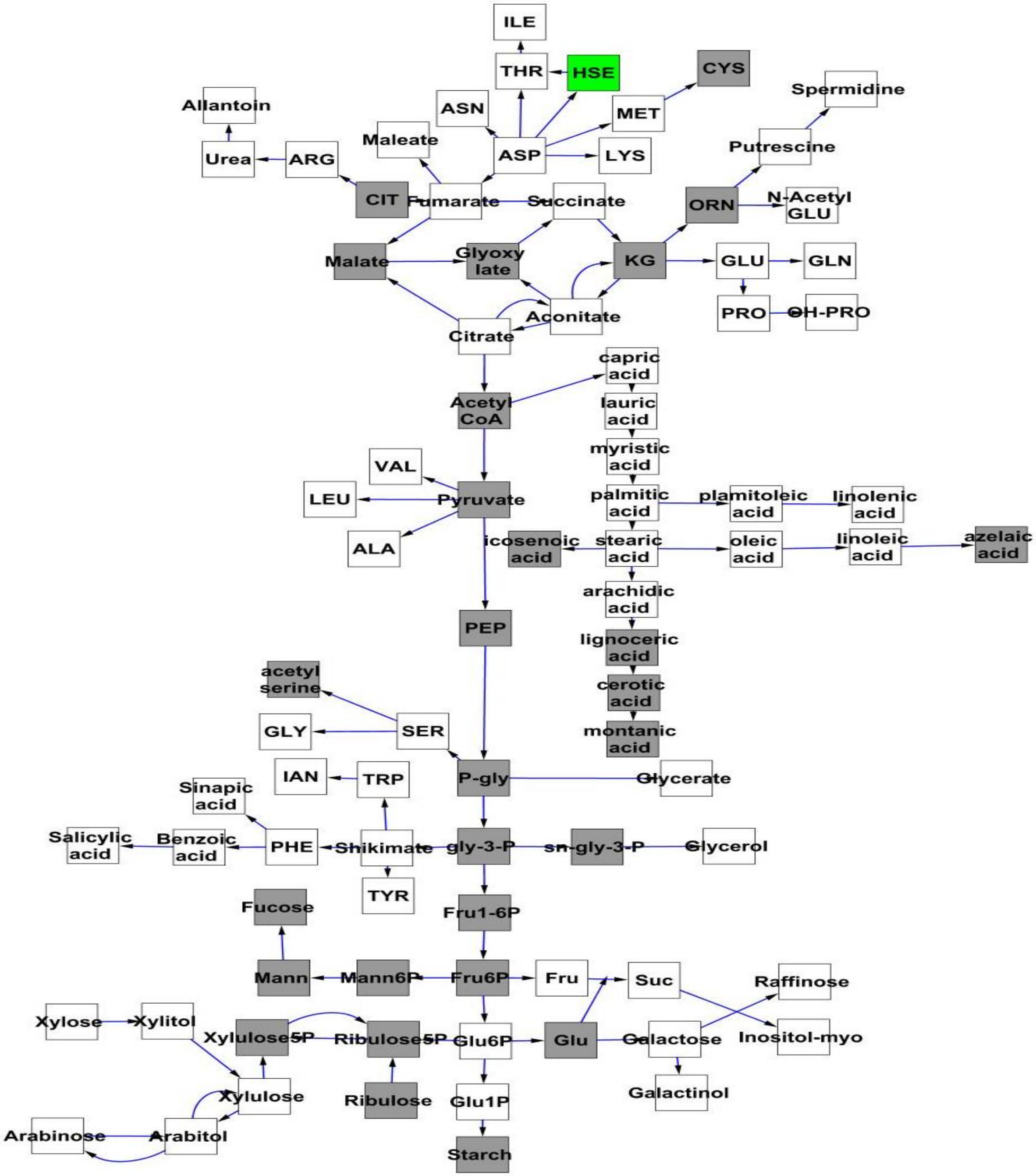

ILE
THR
HSE
CYS
ASN
MET
Spermidine
Allantoin
Maleate
ASP
LYS
Putrescine
Urea
ARG
CIT
Fumarate
Succinate
ORN
N-Acetyl GLU
Malate
Glyoxy late
KG
GLU
GLN
Aconitate
PRO
OH-PRO
Citrate
capric acid
Acetyl CoA
lauric acid
myristic acid
VAL
LEU
Pyruvate
palmitic acid
plamitoleic acid
linolenic acid
ALA
icosenoic acid
stearic acid
oleic acid
linoleic acid
azelaic acid
arachidic acid
PEP
lignoceric acid
acetyl serine
cerotic acid
GLY
SER
montanic acid
IAN
TRP
P-gly
Glycerate
Sinapic acid
Salicylic acid
Benzoic acid
PHE
Shikimate
gly-3-P
sn-gly-3-P
Glycerol
TYR
Fucose
Fru1-6P
Mann
Mann6P
Fru6P
Fru
Suc
Raffinose
Xylose
Xylitol
Xylulose5P
Ribulose5P
Glu6P
Glu
Galactose
Inositol-myo
Xylulose
Ribulose
Glu1P
Galactinol
Arabinose
Arabitol
Starch
